# A framework for macroscopic phase-resetting curves for generalised spiking neural networks

**DOI:** 10.1371/journal.pcbi.1010363

**Published:** 2022-08-01

**Authors:** Grégory Dumont, Alberto Pérez-Cervera, Boris Gutkin

**Affiliations:** 1 Group for Neural Theory, LNC INSERM U960, DEC, Ecole Normale Supérieure - PSL University, Paris France; 2 Center for Cognition and Decision Making, Institute for Cognitive Neuroscience, National Research University Higher School of Economics, Moscow; 3 Instituto de Matemática Interdisciplinar, Universidad Complutense de Madrid, Madrid, Spain; University of Pittsburgh, UNITED STATES

## Abstract

Brain rhythms emerge from synchronization among interconnected spiking neurons. Key properties of such rhythms can be gleaned from the phase-resetting curve (PRC). Inferring the PRC and developing a systematic phase reduction theory for large-scale brain rhythms remains an outstanding challenge. Here we present a theoretical framework and methodology to compute the PRC of generic spiking networks with emergent collective oscillations. We adopt a renewal approach where neurons are described by the time since their last action potential, a description that can reproduce the dynamical feature of many cell types. For a sufficiently large number of neurons, the network dynamics are well captured by a continuity equation known as the refractory density equation. We develop an adjoint method for this equation giving a semi-analytical expression of the infinitesimal PRC. We confirm the validity of our framework for specific examples of neural networks. Our theoretical framework can link key biological properties at the individual neuron scale and the macroscopic oscillatory network properties. Beyond spiking networks, the approach is applicable to a broad class of systems that can be described by renewal processes.

## Author summary

The formation of oscillatory neuronal assemblies at the network level has been hypothesized to be fundamental to many cognitive and motor functions. One prominent tool to understand the dynamics of oscillatory activity response to stimuli, and hence the neural code for which it is a substrate, is a nonlinear measure called Phase-Resetting Curve (PRC). At the network scale, the PRC defines the measure of how a given synaptic input perturbs the timing of next upcoming volley of spike assemblies: either advancing or delaying this timing. As a further application, one can use PRCs to make unambiguous predictions about whether communicating networks of neurons will phase-lock as it is often observed across the cortical areas and what would be this stable phase-configuration: synchronous, asynchronous or with asymmetric phase-shifts. The latter configuration also implies a preferential flow of information form the leading network to the follower, thereby giving causal signatures of directed functional connectivity. Because of the key position of the PRC in studying synchrony, information flow and entrainment to external forcing, it is crucial to move toward a theory that allows to compute the PRCs of network-wide oscillations not only for a restricted class of models, as has been done in the past, but to network descriptions that are generalized and can reflect flexibly single cell properties. In this manuscript, we tackle this issue by showing how the PRC for network oscillations can be computed using the adjoint systems of partial differential equations that define the dynamics of the neural activity density.

This is a *PLOS Computational Biology* Methods paper.

## Introduction

The phase-resetting curve (PRC), popularized by Arthur T. Winfree in 1980 [1], is one of the central tools to study properties and mechanisms of biological rhythms. The PRC is a measure that tracks down the phase shift of a rhythm when a transient perturbation is presented at a determined phase of the oscillatory cycle. The PRC is particularly well adapted to clarify essential dynamical features across a variety of biological contexts [2, 3]. For instance, it has proven to be especially efficient to predict the phase-locking behavior of coupled neural oscillators [4] and rhythms emergent in neural populations [5], to study information flow in networks of bio-chemical oscillators [6], to illustrate the impact of neuromodulation in single neurons experimentally [7] and has been a key classical technique in chronobiology [8].

For oscillatory systems described by ordinary differential equations, the adjoint method provides an accurate procedure to compute the so-called infinitesimal PRC (iPRC) [9]. In the case of vanishingly small perturbation amplitudes, PRC and iPRC become proportional to each other, and therefore, any oscillating dynamical system can be reduced to a single phase equation:

$$\begin{array}{c} \begin{array}{c} \frac{d}{dt}\theta \left(t\right)=\omega +Z\left(\theta \left(t\right)\right)\cdot p\left(t\right). \end{array} \end{array}$$

Here *θ* is the oscillation phase, *ω* is the natural frequency of the oscillator, *p*(*t*) represents the time dependent-perturbation, and the function *Z* the iPRC.

Data suggest that most cortical rhythms emerge from the interactions of irregular spiking cells [10, 11]. Thus the brain oscillatory’s activity results from synchronisation among firing events of large neuronal populations. So far, deriving elementary dynamical systems for such macroscopic oscillation could not be done without drastic simplifications of the individual neurons. From now on, to avoid confusion, we term macroscopic PRC (mPRC), the PRC extracted for an oscillation emerging at the network scale. Initial attempts to derive mPRCs for emergent oscillations [12, 13] required quadratic integrate-and-fire models to describe the neurons. As a consequence, extracting the PRCs of realistic oscillating spiking networks has remained elusive despite its relevance to study brain rhythms [14].

In this paper, we tackle this issue adopting a mean-field description of networks where a given cell is characterized by the amount of time passed by since its last action potential, i.e. the age of the cell.

Originating from the beginning of the 20th century with the paper of Sharpe and Lotka in 1911 [15] and the work of McKendrick in 1926 [16], the study of population dynamics with an age-structured modeling approach has never lost interest within the scientific community. Such models track the time evolution of ages of single individuals and are very well adapted to capture the essential dynamical features of actual data in a wide variety of biological context. They have proven to be especially effective in epidemiology [17–19], cellular proliferation [20–22] and population dynamics [23, 24].

Among the different ways of formulating the problem stands out the von Foerster equation; a continuity equation named after the Austrian American physicist Heinz von Foerster [25]. Written in the form of a partial differential equation, the von Foerster formalism has the tremendous advantage of entailing the other age-structured formulations. So its use is nowadays widespread and favored by theoreticians. The interested reader may find several textbooks in mathematical biology that dedicate a chapter to it [26–28].

Applied to neural systems, this continuity equation is known as the refractory density equation. It was first implemented by Wulfram Gerstner and Leo van Hemmen in 1992 [29]. The refractory equation can rigorously be derived starting from the stochastic process [30], and is amenable to mathematical analysis [31]. Moreover, this continuity equation has been a major tool for studying emergent synchronized assemblies [32], transient dynamics [33], low dimensional reduction [34], and finite-size network activity fluctuations [35–38]. We recommend the reader the textbook [39] for an intuitive introduction on the refractory density equation.

The construction of the refractory density equation relies on a mean-field description of spiking networks where a given cell is characterized by the amount of time passed by since its last action potential. There are undoubtedly alternative ways to describe neurons, however, such a formalism is general as it can effectively reflect many spiking formulations. For instance, renewal processes such as the noisy integrate-and-fire [40–42], or spike response models [32], can be expressed within this framework. Furthermore, this approach provides approximation schemes for complex biophysically-realistic models [43, 44], for correlated noise [45], generalized linear models [46], and for neural adaptation [35, 48], see [48] for a recent review. As a consequence, the refractory density equation can be seen as a general description of spiking neural networks.

This paper is organized as follows. First, we present the network and neuron model that will be used throughout. Then, we obtain the adjoint system which gives access to the PRC. We finish the paper by illustrating a possible application of our framework by studying macroscopic phase locking.

## Results

### Spiking and mean-field description

To describe spiking neurons as renewal processes we need to take into account *h*(*t*), the total input a neuron receives and *r*, the time since the last action potential. Denoting *S*(*h*(*t*), *r*) the escape rate, then, the probability that a firing event occurs during a time interval *dt* is given by *S*(*h*(*t*), *r*)*dt*. Note that the escape rate reflects the individual properties of neurons, as an example, we take an escape rate that captures the dynamics of pyramidal cells [39]. As soon as an action potential is triggered, the neuron’s age *r* is reset to zero. The population activity can be extracted and is given by the sum of all the occurring spikes:

$$\begin{array}{c} A_{N}\left(t\right)=\frac{1}{N}\sum_{k=1}^{N}\sum_{f}\delta \left(t-t_{k}^{f}\right). \end{array}$$ (1)

where *δ* is the Dirac mass, *N* the number of neurons and $t_{k}^{f}$ the firing time of the cell numbered *k*. The total input current is given by

$$\begin{array}{c} \begin{array}{c} h\left(t\right)=I_{ext}\left(t\right)+I_{s}\left(t\right), \end{array} \end{array}$$

where *I*_*ext*_(*t*) is an external current and the synaptic *I*_*s*_(*t*), which defines the current feedback of the network, is given by

$$\begin{array}{c} \begin{array}{c} I_{s}\left(t\right)=J_{s}\kappa *A_{N}\left(t\right)\;\text{with}\;\kappa \left(t\right)=\frac{e^{-t/\tau_{s}}}{\tau_{s}}, \end{array} \end{array}$$

here *J*_*s*_ is the synaptic efficiency, *κ* the normalized synaptic filter and *τ*_*s*_ the synaptic decay.

In the limit of an infinitely large number of neurons *N* (the thermodynamic limit), the full network description reduces to a single partial differential equation. Denoting *q*(*t*, *r*) the probability density for a neuron to have at time *t* an age *r*, the density profile evolves according to the continuity equation:

$$\begin{array}{c} \begin{array}{c} \frac{\partial }{\partial t}q\left(t,r\right)+\frac{\partial }{\partial r}q\left(t,r\right)=-S\left(h\left(t\right),r\right)q\left(t,r\right). \end{array} \end{array}$$ (2)

Because once a cell emits an action potential its age is reset to zero, the natural boundary condition is

$$\begin{array}{c} q(t,0)=A(t), \end{array}$$

where *A*(*t*) is the neural network activity and is defined as

$$\begin{array}{c} A\left(t\right)=\int_{0}^{+\infty }S\left(h\left(t\right),r\right)q\left(t,r\right)\;dr. \end{array}$$ (3)

We recall that in the thermodynamic limit the total input current is given by

$$\begin{array}{c} \begin{array}{c} h\left(t\right)=I_{ext}\left(t\right)+I_{s}\left(t\right)\;\text{with}\;I_{s}\left(t\right)=J_{s}\kappa *A\left(t\right). \end{array} \end{array}$$

**Fig 1:**
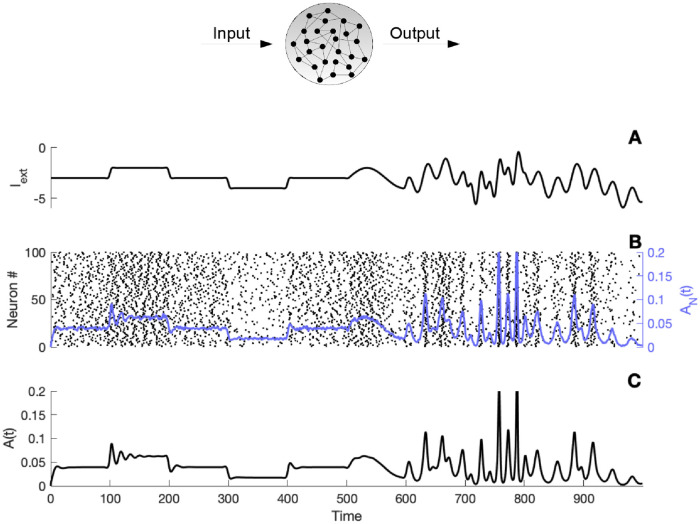
Dynamics for a recurrent excitatory network. Comparison of firing activity. A) Time evolution of the stimulus *I*_*ext*_(*t*). B) Raster plot of 100 neurons, the blue line displays the resulting firing activity Eq (1) of the full network. C) Firing activity obtained from a simulation of the mean-field Eq (3). The simulation was initiated with a similar Gaussian profile for the full network and the mean-field equation, parameters: *S*(*h*, *r*) = exp(*h*)*H*(*r* − *T*_*ref*_) (1 − exp(−(*r* − *T*_*ref*_) /*τ*)), *T*_*ref*_ = 10 *ms*, *τ*_*s*_ = 10 *ms*, *τ* = 5 *ms*, *J*_*s*_ = 15 *mV*.*ms*, *N* = 5000 and Δ*t* = 0.05 *ms*.

The mean-field Eq (2), also termed the von Foerster equation in Mathematical Biology [25], defines a conservation law and expresses three different processes taking place at the cellular level: a drift process due to the time passing between action potentials, an escape rate generated by the randomness of firing events and the individual cell properties, a non-local boundary condition which describes the reset of the neurons that just fired. As we illustrate in Fig 1, the essential shape of the full network activity is well captured by the mean-field Eq (3).

### Emergent macroscopic oscillatory dynamics

to investigate the emergence of macroscopic oscillations we analyze the refractory density Eq (2). after algebraic manipulations—see method for details—we find that the mean activity in the asynchronous regime *a*_∞_ and the mean input *h*_∞_ are given by

$$\begin{array}{c} \begin{array}{c} A_{\infty }^{-1}=\int_{0}^{+\infty }e^{-\int_{0}^{r}S_{\infty }\left(s\right)\;ds}\;dr,\;h_{\infty }=I_{ext}+J_{s}A_{\infty }, \end{array} \end{array}$$ (4)

note that we have used the notation:

$$\begin{array}{c} \begin{array}{c} S_{\infty }\left(r\right)≔S\left(h_{\infty },r\right). \end{array} \end{array}$$

Linearizing around the steady state we extract the characteristic equation, whose solutions give the eigenvalues of the linearized operator, see also [32]. The time-independent solution loses stability and an oscillatory limit cycle gains stability as soon as an eigenvalue has a positive real part. The characteristic equation reads

$$\begin{array}{c} \begin{array}{cc} C(\lambda ) & =J_{s}{\hat{\kappa }}_{\lambda }\int_{0}^{\infty }S_{\infty }\int_{0}^{r}\frac{\partial S_{\infty }}{\partial h}q_{\infty }e^{-\int_{x}^{r}S_{\infty }+\lambda \;ds}\;dx\;dr\\ & +1-J_{s}{\hat{\kappa }}_{\lambda }\int_{0}^{\infty }\frac{\partial S_{\infty }}{\partial h}q_{\infty }\;dr-\int_{0}^{\infty }S_{\infty }e^{-\int_{0}^{r}S_{\infty }+\lambda \;ds}\;dr \end{array} \end{array}$$

where $\;{\hat{\kappa }}_{\lambda }$ is the Laplace transform of the synaptic filter *κ* and *q*_∞_ the steady density profile, see Method for details.

The bifurcation line, which separates an oscillatory dynamic from an asynchronous steady-state regime, can be obtained numerically by solving:

$$\begin{array}{c} C(i\omega )=0. \end{array}$$

**Fig 2:**
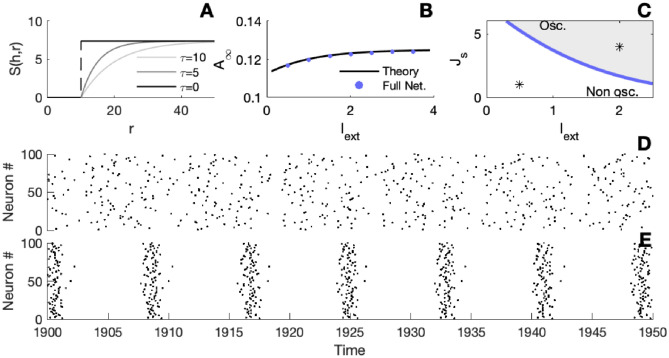
Emergent oscillations. A) Illustration of the escape rate *S*(*h*, *r*) for different values of the parameter *τ*, (*h* = 2 *mV*). B) Comparison between the steady state firing activities with *J*_*s*_ = 1 *mV*.*ms*, blue dots for the full network, and the black line for the theoretical prediction given by (4). C) Bifurcation line in the parameter space (blue curve). The grey shaded region corresponds to an oscillatory regime of the neural network, the white region corresponds to a stable asynchronous mode of the network. D) and E) Raster plots of the spiking activity of 100 neurons. Panel D corresponds to the black asterisk lying in the asynchronous (white) region of panel C, whereas panel E depicts the activity that corresponds to the black asterisk lying in the oscillatory (grey) region of panel C. parameters: *S*(*h*, *r*) = exp(*h*)*H*(*r* − *T*_*ref*_) (1 − exp(−(*r* − *T*_*ref*_) /*τ*)), *T*_*ref*_ = 8 *ms*, *τ*_*s*_ = 10 *ms*, *τ* = 0 *ms*, *N* = 5000 and Δ*t* = 0.1 *ms*.

As we can see from Fig 2C, for a sufficiently large synaptic strength *J*_*s*_ and external current *I*_*ext*_, the asynchronous state undergoes a bifurcation toward oscillations. The simulated spiking activity of the full network in Fig 2D and 2E confirms the emergence of a transition from an asynchronous to a synchronized activity regime when parameters are taken below or above the bifurcation line.

Note that in Fig 2C, the stability line is only found for *τ* = 0. Indeed, in this case, the characteristic equation reduces to a simpler equation which can be solved numerically. We find that taking *τ* to be non-zero affects the position of the stability line. The parameter *τ* plays the role of an effective noise level and the bigger *τ* is, the more current *I*_*ext*_ and/or larger synaptic strength *J*_*s*_ is required to induce oscillations.

Finally, let us emphasize that the observed oscillation is an emergent feature of the network. Individual cells being described by stochastic processes, they cannot produce a regular, i.e. periodic, firing activity. However, at the network level, a self-sustained oscillation emerges, see [49] for another approach on neural syncronization. The oscillation properties can be characterized by the PRC. Such a measure relies on the assumption that additional perturbations are weak enough.

### Phase resetting curve and adjoint method

**Fig 3:**
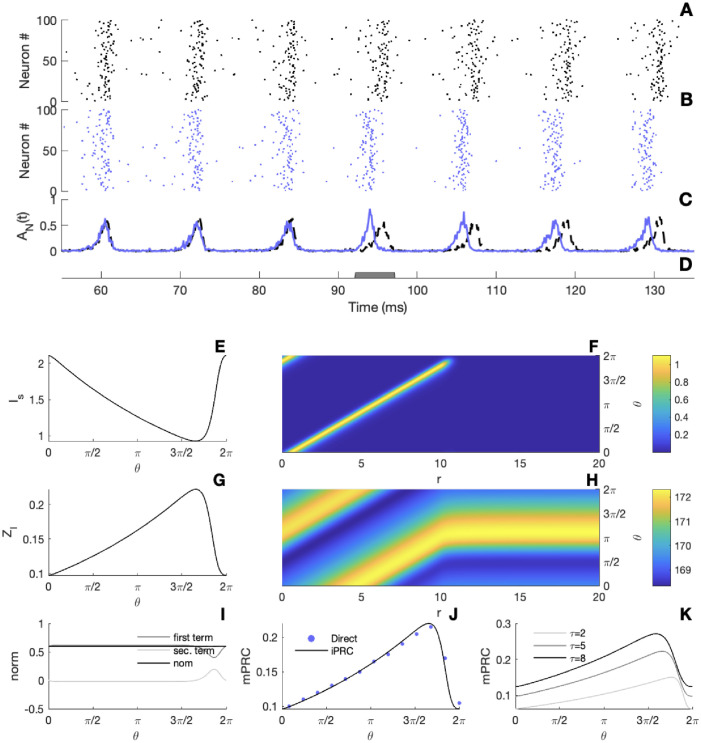
Macroscopic phase-resetting curve. A-B) Raster plot of 100 neurons from a simulation of a non-perturbed/perturbed network. C) Resulting firing activity of the networks obtained from Eq (1), the dashed line in black for the non-perturbed network and full line in blue for the perturbed one. D) Illustration of the stimulus. E) The panel gives the periodic solution of the synaptic current *I*_*s*_(*t*) extracted from the mean-field Eq (2). F) Illustration of the periodic solution of the density function *q*(*t*, *r*) obtained by solving the mean-field Eq (2). G) The panel gives the periodic solution of the first component of the adjoint system (5). H) The panel illustrates the periodic solution of the adjoint density function *Z*_*q*_(*t*, *r*) obtained via (5). I) Illustration of normalizing condition (7). J) The network PRC, the black line illustrates the solution of Eq (5), while blue dots indicate the PRC obtained via direct perturbations. K) Solution of the mPRC (6) for different values of the parameter *τ*. Parameters: *S*(*h*, *r*) = exp(*h*)*H*(*r* − *T*_*ref*_) (1 − exp(−(*r* − *T*_*ref*_) /*τ*)), *I*_*ext*_ = 2 *mV*, *T*_*ref*_ = 10 *ms*, *τ*_*s*_ = 10 *ms*, *τ* = 5 *ms*, *J*_*s*_ = 15 *mV*.*ms*, *N* = 5000 and Δ*t* = 0.05 *ms*. Direct perturbations in panel D were made with a square wave current pulse (amplitude 3 *mV*, duration 5 *ms*) on the full network, and in panel J with a square wave (amplitude 8 *ms*, duration 0.8 *mV*) on the mean-field system (2).

When a brief depolarizing current is applied to the oscillatory network, the global firing activity shifts in time (see Fig 3A–3C). Having the network in an oscillatory regime, that is, having a periodic solution $\left(\bar{q},{\bar{I}}_{s}\right)$ of (2), we find (see Method for details) the mPRC as the solution of the mean-field adjoint equation:

$$\begin{array}{c} -\frac{\partial }{\partial t}Z_{q}\left(t,r\right)-\frac{\partial }{\partial r}Z_{q}\left(t,r\right)=-S\left(\bar{h}\left(t\right),r\right)(Z_{q}\left(t,r\right)-Z_{q}\left(t,0\right)-\frac{J_{s}}{\tau_{s}}Z_{I_{s}}\left(t\right)), \end{array}$$ (5)

and

$$\begin{array}{c} -\frac{d}{dt}Z_{I_{s}}\left(t\right)=-\frac{Z_{I_{s}}\left(t\right)}{\tau_{s}}-\int_{0}^{\infty }(Z_{q}\left(t,r\right)-Z_{q}\left(t,0\right)-\frac{J_{s}}{\tau_{s}}Z_{I_{s}}\left(t\right))\frac{\partial S}{\partial h}\left(\bar{h}\left(t\right),r\right)\bar{q}\left(t,r\right)\;dr, \end{array}$$ (6)

satisfying the normalisation condition

$$\begin{array}{c} \int_{0}^{+\infty }Z_{q}\left(t,r\right)\frac{\partial }{\partial t}\bar{q}\left(t,r\right)\;dr+Z_{I_{s}}\left(t\right)\frac{d}{dt}{\bar{I}}_{s}\left(t\right)=\frac{2\pi }{T}, \end{array}$$ (7)

where *T* is the oscillation period. Note that we made used of the following notation:

$$\begin{array}{c} \bar{h}\left(t\right)=I_{ext}+{\bar{I}}_{s}\left(t\right). \end{array}$$

Although we obtain two functions from the adjoint method, *Z*_*q*_ and $Z_{I_{s}}$, since incoming perturbations come through the synapses, $Z_{I_{s}}$ should be interpreted as the mPRC of the macroscopic oscillation. In Fig 3E–3H, we show an example of a periodic solution and its associated periodic adjoint. The adjoint solution is normalized according to (7), see Fig 3I. We note that the analytically determined mPRC agrees with a PRC obtained from direct perturbations of the spiking network (see in Fig 3J); both are type I. Note that the PRC depends on cell properties, for instance, changing parameters of *S*, e.g. the strength of intrinsic noise (“softness” *τ* of threshold), gives a higher mPRC amplitude as illustrated in Fig 3K, which in turn can impact the locking behavior of multi-network rhythms [5].

Note that, as stated in the introduction, the PRC is a general measure that can be applied to any oscillating dynamical system. For instance, it has been defined for regular spiking cells producing periodic fining and has been argued to reflect the single neurons’ intrinsic excitability properties [50]. In this contribution we computed a macroscopic PRC for the oscillations emerging at the network level. In this setting, individual cells within the network do not have to be all oscillators (with a significant proportion being excitable), and so, the PRC of an individual neuron may not be defined. Indeed, in our networks we have considered stochastic cells forced to fire by random noise which do not have a periodic firing. However, at the network scale, an oscillation emerges from the interaction of irregular spiking activity. We term the PRC computed for the network as the macroscopic PRC (mPRC).

## ISI density and hazard rate

In this section, we briefly recall how to construct the hazard rate function *S*(*h*, *r*) for neurons modeled as time-dependent renewal processes. The class of renewal processes is a wide class of neuron models. Interestingly, it can also be constructed using the interspike interval (ISI) density. We also show how the hazard rate function can be related to the PRC and define its characteristics.

The estimation of the ISI density from experimental data is indeed very common. The interval distribution can be interpreted as a conditional probability density. It is the probability that the next spike occurs in the interval (*t*, *t* + *dt*) given that the last spike occurred at time zero. The hazard rate, also called age-dependent death rate or hazard has the following interpretation that, in order to emit a spike at time *r*, the neuron has to “survive” without firing during the time interval (0, *r*) and then fire at a time *r*. The hazard rate can be determined from the ISI density and its expression is known for decades, see for instance [39]:

$$\begin{array}{c} S\left(h,r\right)=\frac{ISI(h,r)}{1-\int_{0}^{r}ISI\left(h,s\right)ds}, \end{array}$$

which can also be written as:

$$\begin{array}{c} ISI\left(h,r\right)=S\left(h,r\right)e^{-\int_{0}^{r}S\left(h,s\right)ds}. \end{array}$$

Given the interdependence of the hazard rate and the ISI density, one of these functions suffices to apply our theoretical finding exposed in the previous sections and fully determine the PRC. It only requires to have the numerical solutions of *q*(*t*, *r*) and *I*_*s*_(*t*) along the oscillatory cycle, and the expression of the derivative of the hazard rate:

$$\begin{array}{c} \frac{\partial }{\partial h}S\left(h,r\right)=\frac{\frac{\partial }{\partial h}ISI\left(h,r\right)(1-\int_{0}^{r}ISI\left(h,s\right)ds\;)+ISI\left(h,r\right)\int_{0}^{r}\frac{\partial }{\partial h}ISI\left(h,s\right)ds}{{\left(1-\int_{0}^{r}ISI\left(h,s\right)ds\right)}^{2}}. \end{array}$$

The only assumption underlying our methodology to compute the phase-resetting curve for collective rhythms is to have renewal-type spiking neurons.

The difficulty resides in the expression of S and its derivative which are written as quotient. In practice, it can be hard to express numerically the values of the hazard rate and its derivative. It is for instance the case for a popular model widely used in theoretical neuroscience—the leaky integrate-and-fire (LIF) neuron model. Although the ISI density function of the noisy LIF is known, it can be written as a Volterra integral or as an inverse Laplace transform of hypergeometric functions. In practice, it becomes difficult to implement numerically an expression of S and its derivative. Note that the difficulty is numerical and not theoretical.

Another difficult example to deal with is the gamma function. Often used in the literature, the gamma function is known to provide a good fit to the ISI distribution of actual data. It is given by:

$$\begin{array}{c} ISI_{\gamma }\left(h,r\right)=\frac{e^{h\alpha }}{(\alpha -1)!}r^{\alpha -1}e^{-re^{h}}. \end{array}$$

Once again, having the expression of the ISI distribution is sufficient to determine the hazard rate function and from there apply our finding to extract the PRC. However, numerical simulations become tricky and the computation of the derivative of the hazard rate is very unstable. Once again, the difficulty is numerical and not theoretical.

**Fig 4:**
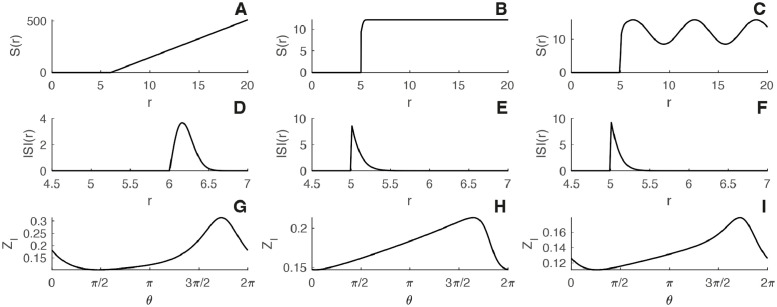
Macroscopic phase-resetting curve and hazard rate. The figure gives the hazard rate function (top panels) together with the resulting mPRC for an excitatory network (bottom panels). A-C) Hazard rate functions. D-F) ISI densities. G-I) Solution of the mPRC (6). Parameters: A-D-G) *S*(*h*, *r*) = exp(*h*)*H*(*r* − *T*_*ref*_)*ε*(*r* − *T*_*ref*_)), *I*_*ext*_ = 2.5 *mV*, *T*_*ref*_ = 6 *ms*, *τ*_*s*_ = 10 *ms*, *J*_*s*_ = 4 *mV*.*ms*, *ε* = 3 and Δ*t* = 0.05 *ms*. B-E-H) *S*(*h*, *r*) = exp(*h*)*H*(*r* − *T*_*ref*_) tanh(exp(*h*)(*r* − *T*_*ref*_)), *I*_*ext*_ = 2.5 *mV*, *T*_*ref*_ = 5 *ms*, *τ*_*s*_ = 10 *ms*, *J*_*s*_ = 3 *mV*.*ms* and Δ*t* = 0.05 *ms*. C-F-I) *S*(*h*, *r*) = exp(*h*)*H*(*r* − *T*_*ref*_) tanh(exp(*h*)(*r* − *T*_*ref*_)) (1 + *ε* cos(*ωr*)), *I*_*ext*_ = 2.5 *mV*, *T*_*ref*_ = 10 *ms*, *τ*_*s*_ = 3 *ms*, *τ* = 5 *ms*, *J*_*s*_ = 15 *mV*.*ms*,*ε* = 3,*ω* = 1 and Δ*t* = 0.05 *ms*. On panels A-B-C) and D-E-F) the hazard rate function and corresponding ISI densities are plotted for *h* = *I*_*ext*_.

In Fig 4 we present several examples used in textbooks to model ISIs or hazard rates of actual neurons [39]. The hazard rate is shown together with the mPRC extracted from our adjoint theory. To compare with results obtained in the previous section, the network is chosen to be purely excitatory. As we can see from the extracted mPRC, the hazard rate function—or the ISI distribution—of the neuron shapes the mPRC differently. This allows to link dynamics of individual neurons, cell type, and the network connectivity to the properties of emerging oscillations at the network scale. Indeed, single cell dynamics clearly have an impact on the macroscopic synchronization properties of the networks. This has been shown in numerous studies. For example, seminal results in [51] showed, using weakly coupled oscillator analysis, how the intrinsic properties of the neurons impact their synchronization properties. It was also shown that the macroscopic oscillation can be shaped by the ISI distribution of single cells [32]. These single cell properties are well documented to be reflected in the hazard function or the ISI distribution [34, 46] and hence a link can be drawn between these and global synchronization of such diverse neurons, see for instance [35] for the role of adaptation.

### The phase equation and emerging locking modes

We now illustrate how the PRC can be used to investigate the dynamical emergence of phase locking states between oscillatory spiking circuits. Such an analysis relies on the assumption that synaptic interactions across networks remain sufficiently weak and that the connection between circuits is fully symmetric. Such an assumption, which guarantees that the perturbed macroscopic oscillations remain close to the unperturbed oscillation, allows us to place our study within the framework of weakly coupled oscillators [2, 3]. We emphasize that within each circuit, neurons are not weakly coupled. The assumption of weak coupling is only made upon the projection across circuits. Within the weakly coupled framework, see [2, 3] for instance, the bidirectionally delayed-coupled neural circuits reduce to a single phase equation (see Method for details):

$$\begin{array}{c} \frac{d}{dt}\theta \left(t\right)=G\left(\theta \left(t\right)\right), \end{array}$$

where *θ*(*t*) is the phase lag—or the phase difference—between circuits and the *G*-function the odd part of the shifted interaction function (see [2, 3]):

$$\begin{array}{c} G(\theta )=H(\theta -d)-H(-\theta -d). \end{array}$$

**Fig 5:**
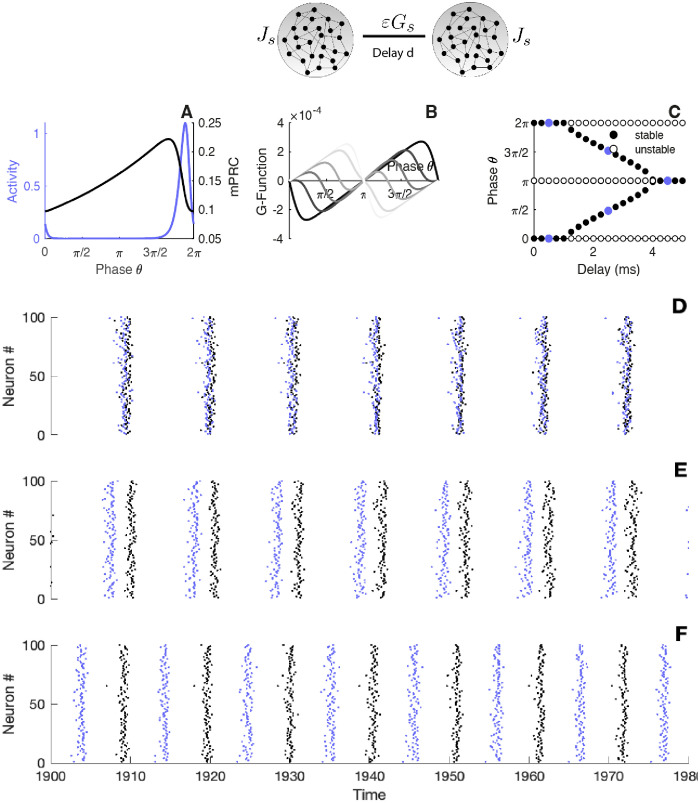
Locking modes of interacting circuits. Top panel: illustration of the two circuits in interaction, *εG*_*s*_ represents the coupling strength across networks, *J*_*s*_ represents the internal coupling strength, *d* represents the delay across circuits. A) The panel displays one period of the activity as well as the mPRC. B) The *G*-function for different parameter values of the delay, dark/light colors correspond to small/large delay. C) Zeros of the *G*-function for different parameter values of the delays. The circles are filled for stable fixed point and empty for the unstable points. D-E-F) Raster plot of the spiking activity of the two neural networks, black dots indicate the spike timing of the first network, coloured dots indicate the spike timing of the second network. Parameters: *S*(*h*, *r*) = exp(*h*)*H*(*r* − *T*_*ref*_) (1 − exp(−(*r* − *T*_*ref*_) /*τ*)), *I*_*ext*_ = 2 *mV*, *T*_*ref*_ = 10 *ms*, *τ*_*s*_ = 10 *ms*, *τ* = 5 *ms*, *J*_*s*_ = 15 *mV*.*ms*, *N* = 5000 *G*_*s*_ = 0.2 *mV*.*ms*, Δ*t* = 0.005 *ms* for all the panels and D) *d* = 0.5 *ms*, E) *d* = 2.5 *ms*, F) *d* = 4.5 *ms*.

Here *d* is the conduction delay between circuits and the interaction function *H* is given by:

$$\begin{array}{c} \begin{array}{cc} H(\theta )= & \frac{\varepsilon G_{s}}{T}\int_{0}^{T}Z_{I}\left(s\right)A\left(s-\theta \right)\;ds \end{array} \end{array}$$

where *T* is the oscillation period, *εG*_*s*_ denotes the connectivity strength between circuits, see Fig 5, and the activity *A*(*t*) in the equation is defined as the activity of one isolated circuit along the oscillatory cycle. Note that the coefficient *ε* is here to emphasize the weak coupling across circuits.

Studying the emergence of a particular locking mode can be done by looking at the zeros of the *G*-function. Each zero of the *G*-function corresponds to a steady state phase lag and its stability can be assessed by looking at the sign of the derivative: zero crossings with a negative slope give stable phase-lags.

In Fig 5A, we display the two quantities of importance to compute the interaction function: one period of the activity and the mPRC obtained via the adjoint method. In Fig 5B we plot the resulting *G*-functions for different values of delay. To get a better understanding, we construct the corresponding bifurcation diagram (Fig 5C) which shows the phase mode positions with respect to delay across circuits. While the stability of the in-phase mode is kept for small delays, for larger transmission delays, a switch of stability takes place allowing the emergence of a whole possibility of phase lags, eventually for large enough delay anti-phase solutions become stable. In Fig 5D–5F we validate this theoretical prediction by showing rasters of the spiking circuits that reflects the modulation of the emerging phase lag by the delay.

**Fig 6:**
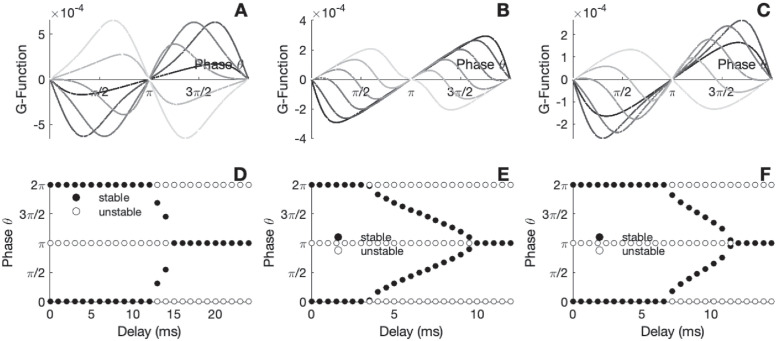
Locking modes of interacting circuits for different ISI densities. A-C) The *G*-function for different parameter values of the delay. D-E) Zeros of the *G*-function for different parameter values of the delays. The circles are filled for stable fixed point and empty for the unstable points. Parameters: A-D) *S*(*h*, *r*) = exp(*h*)*H*(*r* − *T*_*ref*_)*ε*(*r* − *T*_*ref*_)), *I*_*ext*_ = 2.5 *mV*, *T*_*ref*_ = 6 *ms*, *τ*_*s*_ = 10 *ms*, *J*_*s*_ = 4 *mV*.*ms*, *ε* = 3 and Δ*t* = 0.05 *ms*. B-E) *S*(*h*, *r*) = exp(*h*)*H*(*r* − *T*_*ref*_) tanh(exp(*h*)(*r* − *T*_*ref*_)), *I*_*ext*_ = 2.5 *mV*, *T*_*ref*_ = 5 *ms*, *τ*_*s*_ = 10 *ms*, *J*_*s*_ = 3 *mV*.*ms* and Δ*t* = 0.05 *ms*. C-F) *S*(*h*, *r*) = exp(*h*)*H*(*r* − *T*_*ref*_) tanh(exp(*h*)(*r* − *T*_*ref*_)) (1 + *ε* cos(*ωr*)), *I*_*ext*_ = 2.5 *mV*, *T*_*ref*_ = 10 *ms*, *τ*_*s*_ = 3 *ms*, *τ* = 5 *ms*, *J*_*s*_ = 15 *mV*.*ms*,*ε* = 3,*ω* = 1 and Δ*t* = 0.05 *ms*.

In Fig 6, we illustrate how the phase transition is modulated when changing the ISI density of single cells, that is, the individual dynamical feature. It shows how single neuron dynamics (hazard rate/ISI densities) influence macroscopic synchronization properties of connected networks.

## Complementary approach for conductance-based models

In this section, we remind the reader of another use of the renewal framework in Computational Neuroscience. In the seminal work [44], the authors have constructed a particularly relevant mapping between voltage-based models and the renewal equation at the core of this paper, see also [48] for a recent review. For instance, starting with the leaky integrate-and-fire model, see [52]:

$$\begin{array}{c} C\frac{dv}{dt}=-G\left(v-V_{L}\right)+h\left(t\right)+\sigma \eta \left(t\right), \end{array}$$

together with a threshold *V*_*T*_ and a reset *V*_*r*_ to account for the emission of an action potential. Here *h*(*t*) is the total stimulus, *C* is the capacitance, *G*, the conductance, *V*_*L*_, the reversal potential, and *σ* the scaling of the white noise *η*. The authors have shown that this is equivalent to the formulation (see [52]):

$$\begin{array}{c} \begin{array}{c} \frac{\partial }{\partial t}q\left(t,r\right)+\frac{\partial }{\partial r}q\left(t,r\right)=-S\left(u\left(t,r\right),\dot{u}\left(t,r\right)\right)q\left(t,r\right), \end{array} \end{array}$$

where *u*(*t*, *r*) is given by

$$\begin{array}{c} \begin{array}{c} C(\frac{\partial }{\partial t}u\left(t,r\right)+\frac{\partial }{\partial r}u\left(t,r\right))=-G\left(u\left(t,r\right)-V_{L}\right)+h\left(t\right). \end{array} \end{array}$$

The boundary conditions of the two partial differential equations are given by

$$\begin{array}{c} q\left(t,0\right)=\int_{0}^{+\infty }S\left(u\left(t,r\right),\dot{u}\left(t,r\right)\right)q\left(t,r\right)\;dr, \end{array}$$

and for *u*, it is given by

$$\begin{array}{c} u\left(t,0\right)=V_{r}. \end{array}$$

The hazard rate function *S* has been computed for different models, see [48] for a review. It would therefore be extremely interesting to see how to extract the mPRC, that is, to compute the adjoint equation. Of course, simulations would have to be performed to see how the theoretical result compares with simulations. While a full treatment for two coupled partial differential equations is beyond the scope of this paper, our initial computations seem to carry out smoothly: in the appendix we lay out a pathway to compute the adjoint for this description.

## Discussion

Rhythms are ubiquitous in the nervous system e.g., across the cortex as well as within the spinal cord [10]. They reflect synchronized spiking activity of neurons and are classified in frequency bands: delta (0.5–4 Hz), theta (4–10 Hz), alpha (8–12 Hz), beta (10–30 Hz) and gamma (30–100 Hz). Brain oscillations are known to be involved in numerous functions such as perception, motor coordination and cognition. Excess or deficit in oscillations or synchrony may lead to neurological disorders. To better understand the informational properties of neural oscillations, recent experimental studies have made use of numerically compiled neural population PRCs [53] to show how the brain rhythms react to inputs.

Previous efforts to go beyond these numerical compilations to population PRCs required restrictions on the neuronal models used [13]. Notably, in our previous work we computed macroscopic PRCs for exact reduced networks [5, 13] semi-analytically, but this required the single cells to be modelled by the quadratic-integrate-and-fire neurons with Lorentzian heterogeneity. Adjoint methods for wider classes of networks has been an outstanding question to be resolved.

Another recently developed approach deals also with computing phase response curves for infinite dimensional equations, in particular, for drift diffusion systems [12], see also [3] for a review on the subject of drift diffusion and reaction diffusion. However, in neural context, this method is limited in application to diffusion systems with periodic boundary conditions, and therefore allows, once again, to treat only the quadratic integrate-and-fire neuron model with threshold and reset at infinity. Our approach has the advantage to be more general and allows to treat in theory any neuron model belonging to the class of renewal processes.

In this computational methods paper, we develop a theoretical framework to compute the PRC of emergent macroscopic network-wide oscillations in population models described by refractory density equations. Our methodology, here applied to spiking networks of excitatory cells, provides a path to study the links between microscopic cellular excitability properties, the network coupling and the informational properties of the emerging brain rhythms.

Interestingly, recent studies have shown that the refractory framework, known as the von Foerster framework in Mathematical Biology [25], is powerful enough to entail many cell type dynamics. Such a generality of the refractory density approach relies on the quasi-renewal approximation [47]. This approximation was first introduced to include adaptation due to calcium entry after spikes and neurotransmitter release acting over larger time scales. A recent study has shown that quasi-renewal approximation permits the transition from General Linear Model (GLM) to the escape rate function [54]. GLM point-processes being able to encapsulate the dynamical aspects of most single cell types [46], the refractory density equation can serve as paradigmatic model to describe general network activity. Therefore, the methodology presented here can be applied to a wide variety of network models and architectures (see Methods for the generalization of our results to excitatory-inhibitory networks).

Importantly, as we just mentioned, our method is general enough to entail many cell types. Indeed, having the expression of the ISI distribution is sufficient to determine the hazard rate function and from there apply our theoretical finding to extract the mPRC. However, numerical simulations can be tricky and unstable. Let us emphasize that the difficulty is numerical and not theoretical, and therefore our approach is currently limited to neural dynamics having a closed form expression of the hazard rate function. Another current limitation is the simple architecture of the network. Although it is possible to compute the mPRC for an E-I network (see Method), it results in a system of two coupled partial differential equations which might be hard to solve numerically.

To illustrate our theoretical finding, we have studied the macroscopic phase-locking behaviour between two oscillatory circuits. Within the weakly coupled oscillator framework [3], we have illustrated how the mPRC allows us to construct a bifurcation diagram predicting locking modes between circuits depending on relevant parameters such as synaptic delay, connectivity, etc. Further applications could initiate further studies and benefit our understanding of brain oscillations. For instance, PRC can serve the study of entertainment to periodic inputs, coding and information transfer [6, 55, 56]; or, expanding on our previous work [5], to study the impact of cellular properties on the different phase-locking patterns underlying directed signaling and functional connectivity in single and intercoupled oscillatory networks [57–61].

We believe that our approach can be applied widely to intercoupled networks with individual elements whose complexity can be incorporated into the mean-field continuity equations (e.g. cell proliferation [20–22], population dynamics [23, 24], epidemiological models [17–19]). The von-Foerster equation at the core of this paper is indeed a paradigmatic approach employed to study the population dynamics in many different contexts in Computational and Mathematical Biology [26–28].

## Methods

In the subsequent sections, we present the extended details yielding to the derivations of the framework introduced in the main text. The Method section is structured as follows: we remind the refractory density equation. Then give the expression the steady states and its stability properties are discussed. Next, we derive the main result: the adjoint equation giving access to the infinitesimal phase resetting curve of the network. The normalisation condition is presented. Numerical details about the procedure of to solve the adjoint equations are given. We finish describing the extension of our results to excitatory-inhibitory networks.

### Mean-field description

Denoting *q*(*t*, *r*) the probability density for a neuron to have at time *t* an age *r*, the refractory density profile evolves according to the continuity equation:

$$\begin{array}{c} \begin{array}{c} \frac{\partial }{\partial t}q\left(t,r\right)+\frac{\partial }{\partial r}q\left(t,r\right)=-S\left(h\left(t\right),r\right)q\left(t,r\right). \end{array} \end{array}$$ (8)

The function *S*(*h*(*t*), *r*) is the escape rate which reflects the individual properties of neurons. The total input current *h*(*t*) is given by

$$\begin{array}{c} \begin{array}{c} h\left(t\right)=I_{ext}\left(t\right)+I_{s}\left(t\right), \end{array} \end{array}$$

where *I*_*ext*_ is the external current and *I*_*s*_ the synaptic current:

$$\begin{array}{c} \begin{array}{c} I_{s}\left(t\right)=J_{s}\kappa *A\left(t\right). \end{array} \end{array}$$

Here *J*_*s*_ is the synaptic efficiency, *A*(*t*) the firing activity defined as

$$\begin{array}{c} A\left(t\right)=\int_{0}^{+\infty }S\left(h\left(t\right),r\right)q\left(t,r\right)\;dr, \end{array}$$

and *κ* the normalized synaptic filter

$$\begin{array}{c} \begin{array}{c} \kappa \left(t\right)=\frac{e^{-t/\tau_{s}}}{\tau_{s}}, \end{array} \end{array}$$

with *τ*_*s*_ the synaptic decay.

The mean-field Eq (8) is endowed with a boundary condition:

$$\begin{array}{c} q(t,0)=A(t). \end{array}$$

### Steady state

The asynchronous state can be computed as the time independent solution of the refractory density equation. Let us denote *q*_∞_(*r*) the steady state, and *A*_∞_ the mean firing rate. We have the following equation

$$\begin{array}{c} \begin{array}{c} \frac{d}{dr}q_{\infty }\left(r\right)=-S\left(h_{\infty },r\right)q_{\infty }\left(r\right), \end{array} \end{array}$$

where we have noted

$$\begin{array}{c} \begin{array}{c} h_{\infty }=I_{ext}+J_{s}A_{\infty }. \end{array} \end{array}$$

The equation can be integrated and gives us

$$\begin{array}{c} \begin{array}{c} q_{\infty }\left(r\right)=A_{\infty }e^{-\int_{0}^{r}S\left(h_{\infty },s\right)\;ds}, \end{array} \end{array}$$

where we have used the natural boundary condition

$$\begin{array}{c} \begin{array}{c} q_{\infty }\left(0\right)=A_{\infty }. \end{array} \end{array}$$

Finally, the asynchronous mean firing rate can be computed using the conservation property of the neural network

$$\begin{array}{c} \begin{array}{c} \int_{0}^{\infty }q_{\infty }\left(r\right)\;dr=1, \end{array} \end{array}$$

and we get

$$\begin{array}{c} \begin{array}{c} A_{\infty }^{-1}=\int_{0}^{\infty }e^{-\int_{0}^{r}S\left(h_{\infty },s\right)\;ds}\;dr, \end{array} \end{array}$$

Note that the mean firing rate is only implicitly given since *h*_∞_ does depends on *A*_∞_.

With our choices of functions

$$\begin{array}{c} \begin{array}{c} S\left(h_{\infty },r\right)=e^{h_{\infty }}H\left(r-T_{ref}\right), \end{array} \end{array}$$

we can push further the computation, and after algebraic manipulations, we find that the mean firing activity *A*_∞_ is solution of the nonlinear equation

$$\begin{array}{c} \begin{array}{c} A_{\infty }={\left(T_{ref}+e^{-I_{ext}-J_{s}A_{\infty }}\right)}^{-1}, \end{array} \end{array}$$ (9)

which can be solved numerically.

### Stability analysis

To study the stability of the asynchronous state, one needs the eigenvalues of the differential operator once a linearization around the steady state has been performed. We therefore consider a small perturbation and write the solution in the form

$$\begin{array}{c} \begin{array}{c} q\left(t,r\right)=q_{\infty }\left(r\right)+\varepsilon q_{1}\left(t,r\right)+O\left(\varepsilon^{2}\right),\;A\left(t\right)=A_{\infty }+\varepsilon A_{1}\left(t\right)+O\left(\varepsilon^{2}\right). \end{array} \end{array}$$

Plugging these expressions into Eq (8)—keeping the first order terms only—yields the partial differential equation

$$\begin{array}{c} \begin{array}{c} \frac{\partial }{\partial t}q_{1}\left(t,r\right)+\frac{\partial }{\partial r}q_{1}\left(t,r\right)=-S\left(h_{\infty },r\right)q_{1}\left(t,r\right)-J_{s}\frac{\partial S}{\partial h}\left(h_{\infty },r\right)q_{\infty }\left(r\right)\kappa *A_{1}\left(t\right), \end{array} \end{array}$$

and for the activity

$$\begin{array}{c} \begin{array}{c} A_{1}\left(t\right)=\int_{0}^{+\infty }S\left(h_{\infty },r\right)q_{1}\left(t,r\right)\;dr+J_{s}\kappa *A_{1}\left(t\right)\int_{0}^{+\infty }\frac{\partial S}{\partial h}\left(h_{\infty },r\right)q_{\infty }\left(r\right)\;dr. \end{array} \end{array}$$

Since we are interested in the long term behavior of the perturbation we express the perturbation in eigenvalue mode

$$\begin{array}{c} \begin{array}{c} q_{1}\left(t,r\right)=e^{\lambda t}q_{1}\left(r\right),\;A_{1}\left(t\right)=e^{\lambda t}A_{1}. \end{array} \end{array}$$

After algebraic manipulations, we get that the perturbation obeys to

$$\begin{array}{c} \begin{array}{c} \lambda q_{1}\left(r\right)+\frac{d}{dr}q_{1}\left(r\right)=-(S(h_{\infty },r)+\lambda )q_{1}\left(r\right)-J_{s}A_{1}\frac{\partial S}{\partial h}\left(h_{\infty },r\right)q_{\infty }\left(r\right)\hat{\kappa }\left(\lambda \right), \end{array} \end{array}$$

where we have introduced $\hat{\kappa }$ the Laplace transform *κ*:

$$\begin{array}{c} \hat{\kappa }\left(\lambda \right)=\int_{0}^{\infty }\kappa \left(s\right)\;\text{exp}\;\left(-\lambda s\right)\;ds, \end{array}$$

and for the activity

$$\begin{array}{c} \begin{array}{c} A_{1}(1-J_{s}\hat{\kappa }\left(\lambda \right)\int_{0}^{+\infty }\frac{\partial S}{\partial h}\left(h_{\infty },r\right)q_{\infty }\left(r\right)\;dr)=\int_{0}^{+\infty }S\left(h_{\infty },r\right)q_{1}\left(r\right)\;dr. \end{array} \end{array}$$

Integrating this solution with the variation of constants method, we get

$$\begin{array}{c} \begin{array}{c} q_{1}\left(r\right)=A_{1}e^{-\int_{0}^{r}S\left(h_{\infty },s\right)+\lambda \;ds}-J_{s}A_{1}\hat{\kappa }\left(\lambda \right)\int_{0}^{r}\frac{\partial S}{\partial h}\left(h_{\infty },x\right)q_{\infty }\left(x\right)e^{-\int_{x}^{r}S\left(h_{\infty },s\right)+\lambda \;ds}\;dx, \end{array} \end{array}$$

which implies

$$\begin{array}{c} \begin{array}{cc} \int_{0}^{+\infty } & S\left(h_{\infty },r\right)q_{1}\left(r\right)\;dr\\ = & -J_{s}A_{1}\hat{\kappa }\left(\lambda \right)\int_{0}^{+\infty }S\left(h_{\infty },x\right)\int_{0}^{r}\frac{\partial S}{\partial h}\left(h_{\infty },x\right)q_{\infty }\left(x\right)e^{-\int_{x}^{r}S\left(h_{\infty },s\right)+\lambda \;ds}\;dx\;dr\\ & +A_{1}\int_{0}^{+\infty }S\left(h_{\infty },r\right)e^{-\int_{0}^{r}S\left(h_{\infty },s\right)+\lambda \;ds}\;dr, \end{array} \end{array}$$

and we finally arrive on the equation

$$\begin{array}{c} \;1-J_{s}\hat{\kappa }\left(\lambda \right)\int_{0}^{+\infty }\frac{\partial S}{\partial h}\left(h_{\infty },r\right)q_{\infty }\left(r\right)\;dr\\ +J_{s}\hat{\kappa }\left(\lambda \right)\int_{0}^{+\infty }S\left(h_{\infty },r\right)\int_{0}^{r}\frac{\partial S}{\partial h}\left(h_{\infty },x\right)q_{\infty }\left(x\right)e^{-\int_{x}^{r}S\left(h_{\infty },s\right)+\lambda \;ds}\;dx\;dr\\ \;-\int_{0}^{+\infty }S\left(h_{\infty },r\right)e^{-\int_{0}^{r}S\left(h_{\infty },s\right)+\lambda \;ds}\;dr=0. \end{array}$$

We therefore write down the characteristic equation of the eigenvalues as

$$\begin{array}{c} \begin{array}{cc} C\left(\lambda \right)=1-J_{s}\hat{\kappa }\left(\lambda \right)\int_{0}^{+\infty }\frac{\partial S}{\partial h}\left(h_{\infty },r\right)q_{\infty }\left(r\right)\;dr-\int_{0}^{+\infty }S\left(h_{\infty },r\right)e^{-\int_{0}^{r}S\left(h_{\infty },s\right)+\lambda \;ds}\;dr & \\ +J_{s}\hat{\kappa }\left(\lambda \right)\int_{0}^{+\infty }S\left(h_{\infty },r\right)\int_{0}^{r}\frac{\partial S}{\partial h}\left(h_{\infty },x\right)q_{\infty }\left(x\right)e^{-\int_{x}^{r}S\left(h_{\infty },s\right)+\lambda \;ds}\;dx\;dr & . \end{array} \end{array}$$

With the special choice

$$\begin{array}{c} \begin{array}{c} S\left(h_{\infty },r\right)=e^{h_{\infty }}H\left(r-T_{ref}\right), \end{array} \end{array}$$

we can push further the computation, and after algebraic manipulations, we find:

$$\begin{array}{c} \begin{array}{c} C\left(\lambda \right)=\lambda -J_{s}\lambda \hat{\kappa }\left(\lambda \right)A_{\infty }+e^{h_{\infty }}-e^{h_{\infty }-\lambda T_{ref}}. \end{array} \end{array}$$

The bifurcation line, which separates an oscillatory dynamic from an asynchronous regime, can be obtained numerically by solving

$$\begin{array}{c} \begin{array}{c} C(i\omega )=0. \end{array} \end{array}$$

### The adjoint equation

To compute the PRC, we first rewrite the synaptic filtering as a differential equation. Having

$$\begin{array}{c} \begin{array}{c} I_{s}\left(t\right)=J_{s}\kappa *A\left(t\right),\;\kappa \left(t\right)=\frac{e^{-t/\tau_{s}}}{\tau_{s}}, \end{array} \end{array}$$

is equivalent as having:

$$\begin{array}{c} \begin{array}{c} \tau_{s}\frac{d}{dt}I_{s}\left(t\right)=-I_{s}\left(t\right)+J_{s}A\left(t\right). \end{array} \end{array}$$

We then assume that there is a stable oscillatory solution $\left(q_{o},I_{s_{o}}\right)$ of period *T* for the mean-field equation. Considering a small perturbation around the stable solution, we write

$$\begin{array}{c} \begin{array}{c} q\left(t,r\right)=q_{o}\left(t,r\right)+\varepsilon q_{p}\left(t,r\right)+O\left(\varepsilon^{2}\right),\;I_{s}\left(t\right)=I_{s_{o}}\left(t\right)+\varepsilon I_{s_{p}}\left(t\right)+O\left(\varepsilon^{2}\right). \end{array} \end{array}$$

Plugging these expressions and only keeping the first order term, we get that the perturbation obeys to the following set of equations

$$\begin{array}{c} \begin{array}{c} \frac{\partial }{\partial t}q_{p}\left(t,r\right)+\frac{\partial }{\partial r}q_{p}\left(t,r\right)=-S\left(h_{o}\left(t\right),r\right)q_{p}\left(t,r\right)-\frac{\partial S}{\partial h}\left(h_{o}\left(t\right),r\right)q_{o}\left(t,r\right)I_{s_{p}}\left(t\right), \end{array} \end{array}$$

where

$$\begin{array}{c} \begin{array}{c} h_{o}\left(t\right)=I_{ext}+I_{s_{o}}\left(t\right), \end{array} \end{array}$$

and for the activity

$$\begin{array}{c} \begin{array}{c} A_{p}\left(t\right)=\int_{0}^{+\infty }S\left(h_{o}\left(t\right),r\right)q_{p}\left(t,r\right)\;dr+I_{s_{p}}\left(t\right)\int_{0}^{+\infty }\frac{\partial S}{\partial h}\left(h_{o}\left(t\right),r\right)q_{o}\left(t,r\right)\;dr, \end{array} \end{array}$$

the boundary condition follows as

$$\begin{array}{c} \begin{array}{c} q_{p}\left(t,0\right)=A_{p}\left(t\right), \end{array} \end{array}$$

with

$$\begin{array}{c} \begin{array}{c} \tau_{s}\frac{d}{dt}I_{s_{p}}\left(t\right)=-I_{s_{p}}\left(t\right)+J_{s}A_{p}\left(t\right). \end{array} \end{array}$$

Now, we can define a bilinear form as

$$\begin{array}{c} \langle (\begin{array}{c} q_{1}\\ I_{1} \end{array}),(\begin{array}{c} q_{2}\\ I_{2} \end{array});t\rangle =\int_{0}^{+\infty }q_{1}\left(t,r\right)q_{2}\left(t,r\right)\;dr+I_{1}\left(t\right)I_{2}\left(t\right). \end{array}$$

Different approaches exist to compute the PRC. These have been previously reviewed and the interested reader can look at the textbook [56] as well as at the review on experimental approaches to PRC measurement [62]. The PRC can be computed using a singular perturbation approach or a more geometrical approach relying on isochrons, see [56]. Whereas each approach has its own advantage, both of them are difficult to generalize when it comes to partial differential equations. Interestingly, a very simple method has been proposed relying only on dot products and algebraic computations [9], see also [56] for a review regarding the three different approaches. Namely, we use the fact that the asymptotic phase to an infinitesimal perturbation

$$\left\langle \left(\begin{array}{c} Z_{q}\\ Z_{I_{s}} \end{array}\right),\left(\begin{array}{c} q_{p}\\ I_{p} \end{array}\right)\right\rangle$$

is independent of time for small perturbation *q*_*p*_, *I*_*p*_. We recommend the reader to look at [9, 56] for a mathematical justification. Therefore the PRC (*Z*_*q*_, *Z*_*I*_) would be given by the following property

$$\begin{array}{c} \begin{array}{c} \frac{d}{dt}\langle (\begin{array}{c} Z_{q}\\ Z_{I_{s}} \end{array}),(\begin{array}{c} q_{p}\\ I_{p} \end{array});t\rangle =0. \end{array} \end{array}$$

Developing the first term we get that

$$\begin{array}{c} \begin{array}{c} \frac{d}{dt}\int_{0}^{+\infty }Z_{q}\left(t,r\right)q_{p}\left(t,r\right)\;dr=\int_{0}^{+\infty }q_{p}\left(t,r\right)\frac{\partial }{\partial t}Z_{q}\left(t,r\right)+Z_{q}\left(t,r\right)\frac{\partial }{\partial t}q_{p}\left(t,r\right)\;dr, \end{array} \end{array}$$

and plugging the expression of $\frac{\partial }{\partial t}q_{p}\left(t,r\right)$ inside the equation, we obtain

$$\begin{array}{c} \begin{array}{cc} \frac{d}{dt}\int_{0}^{+\infty } & Z_{q}\left(t,r\right)q_{p}\left(t,r\right)\;dr\\ = & \int_{0}^{+\infty }Z_{q}\left(t,r\right)(-\frac{\partial }{\partial r}q_{p}\left(t,r\right)-S\left(h_{o}\left(t\right),r\right)q_{p}\left(t,r\right)-\frac{\partial S}{\partial h}\left(h_{o}\left(t\right),r\right)q_{o}\left(t,r\right)I_{s_{p}}\left(t\right))\;dr\\ & +\int_{0}^{+\infty }q_{p}\left(t,r\right)\frac{\partial }{\partial t}Z_{q}\left(t,r\right)\;dr, \end{array} \end{array}$$

developing the terms lead to

$$\begin{array}{c} \begin{array}{cc} \frac{d}{dt}\int_{0}^{+\infty } & Z_{q}\left(t,r\right)q_{p}\left(t,r\right)\;dr\\ = & -\int_{0}^{+\infty }Z_{q}\left(t,r\right)\frac{\partial }{\partial r}q_{p}\left(t,r\right)\;dr-\int_{0}^{+\infty }Z_{q}\left(t,r\right)S\left(h_{o}\left(t\right),r\right)q_{p}\left(t,r\right)\;dr\\ & -I_{s_{p}}\left(t\right)\int_{0}^{+\infty }Z_{q}\left(t,r\right)\frac{\partial S}{\partial h}\left(h_{o}\left(t\right),r\right)q_{o}\left(t,r\right)\;dr+\int_{0}^{+\infty }q_{p}\left(t,r\right)\frac{\partial }{\partial t}Z_{q}\left(t,r\right)\;dr. \end{array} \end{array}$$

Applying an integration by parts we get

$$\begin{array}{c} \begin{array}{cc} \int_{0}^{+\infty }Z_{q}\left(t,r\right)\frac{\partial }{\partial r}q_{p}\left(t,r\right)\;dr= & {\left[Z_{q}\left(t,r\right)q_{p}\left(t,r\right)\right]}_{0}^{+\infty }-\int_{0}^{+\infty }\frac{\partial }{\partial r}Z_{q}\left(t,r\right)q_{p}\left(t,r\right)\;dr\\ = & -Z_{q}\left(t,0\right)q_{p}\left(t,0\right)-\int_{0}^{+\infty }\frac{\partial }{\partial r}Z_{q}\left(t,r\right)q_{p}\left(t,r\right)\;dr\\ = & -Z_{q}\left(t,0\right)A_{p}\left(t\right)-\int_{0}^{+\infty }\frac{\partial }{\partial r}Z_{q}\left(t,r\right)q_{p}\left(t,r\right)\;dr. \end{array} \end{array}$$

Therefore we have

$$\begin{array}{c} \begin{array}{c} \frac{d}{dt}\int_{0}^{+\infty }Z_{q}\left(t,r\right)q_{p}\left(t,r\right)\;dr=Z_{q}\left(t,0\right)A_{p}\left(t\right)+\int_{0}^{+\infty }\frac{\partial }{\partial r}Z_{q}\left(t,r\right)q_{p}\left(t,r\right)\;dr\\ -\int_{0}^{+\infty }Z_{q}\left(t,r\right)S\left(h_{o}\left(t\right),r\right)q_{p}\left(t,r\right)\;dr\\ -I_{s_{p}}\left(t\right)\int_{0}^{+\infty }Z_{q}\left(t,r\right)\frac{\partial S}{\partial h}\left(h_{o}\left(t\right),r\right)q_{o}\left(t,r\right)\;dr+\int_{0}^{+\infty }q_{p}\left(t,r\right)\frac{\partial }{\partial t}Z_{q}\left(t,r\right)\;dr, \end{array} \end{array}$$

which is equivalent to

$$\begin{array}{c} \begin{array}{c} \frac{d}{dt}\int_{0}^{+\infty }Z_{q}\left(t,r\right)q_{p}\left(t,r\right)\;dr=\int_{0}^{+\infty }(\frac{\partial }{\partial t}Z_{q}\left(t,r\right)+\frac{\partial }{\partial r}Z_{q}\left(t,r\right)-S\left(h_{o}\left(t\right),r\right)Z_{q}\left(t,r\right))q_{p}\left(t,r\right)\;dr\\ +Z_{q}\left(t,0\right)A_{p}\left(t\right)-I_{s_{p}}\left(t\right)\int_{0}^{+\infty }Z_{q}\left(t,r\right)\frac{\partial S}{\partial h}\left(h_{o}\left(t\right),r\right)q_{o}\left(t,r\right)\;dr, \end{array} \end{array}$$

We now develop the second term

$$\begin{array}{c} \begin{array}{c} \frac{d}{dt}[Z_{I_{s}}\left(t\right)I_{s_{p}}\left(t\right)]=I_{s_{p}}\left(t\right)\frac{d}{dt}Z_{I_{s}}\left(t\right)+Z_{I_{s}}\left(t\right)\frac{d}{dt}I_{s_{p}}\left(t\right), \end{array} \end{array}$$

and recalling the fact that

$$\begin{array}{c} \begin{array}{c} \tau_{s}\frac{d}{dt}I_{s_{p}}\left(t\right)=-I_{s_{p}}\left(t\right)+J_{s}A_{p}\left(t\right), \end{array} \end{array}$$

we obtain

$$\begin{array}{c} \begin{array}{c} \frac{d}{dt}[Z_{I_{s}}\left(t\right)I_{s_{p}}\left(t\right)]=I_{s_{p}}\left(t\right)\frac{d}{dt}Z_{I_{s}}\left(t\right)-\frac{1}{\tau_{s}}Z_{I_{s}}\left(t\right)I_{s_{p}}\left(t\right)+\frac{J_{s}}{\tau_{s}}Z_{I_{s}}\left(t\right)A_{p}\left(t\right). \end{array} \end{array}$$

Now, putting everything together

$$\begin{array}{c} \begin{array}{c} \frac{d}{dt}\langle (\begin{array}{c} Z_{q}\\ Z_{I_{s}} \end{array}),(\begin{array}{c} q_{p}\\ I_{s_{p}} \end{array});t\rangle =\frac{d}{dt}\int_{0}^{+\infty }Z_{q}\left(t,r\right)q_{p}\left(t,r\right)\;dr+\frac{d}{dt}[Z_{I_{s}}\left(t\right)I_{s_{p}}\left(t\right)], \end{array} \end{array}$$

which gives

$$\begin{array}{c} \begin{array}{c} \frac{d}{dt}\langle (\begin{array}{c} Z_{q}\\ Z_{I_{s}} \end{array}),(\begin{array}{c} q_{p}\\ I_{s_{p}} \end{array});t\rangle =\int_{0}^{+\infty }(\frac{\partial }{\partial t}Z_{q}\left(t,r\right)+\frac{\partial }{\partial r}Z_{q}\left(t,r\right)-S\left(h_{o}\left(t\right),r\right)Z_{q}\left(t,r\right))q_{p}\left(t,r\right)\;dr\\ +Z_{q}\left(t,0\right)A_{p}\left(t\right)-I_{s_{p}}\left(t\right)\int_{0}^{+\infty }Z_{q}\left(t,r\right)\frac{\partial S}{\partial h}\left(h_{o}\left(t\right),r\right)q_{o}\left(t,r\right)\;dr\\ +I_{s_{p}}\left(t\right)\frac{d}{dt}Z_{I_{s}}\left(t\right)-\frac{1}{\tau_{s}}Z_{I_{s}}\left(t\right)I_{s_{p}}\left(t\right)+\frac{J_{s}}{\tau_{s}}Z_{I_{s}}\left(t\right)A_{p}\left(t\right). \end{array} \end{array}$$

We now use the fact that

$$\begin{array}{c} \begin{array}{c} A_{p}\left(t\right)=\int_{0}^{+\infty }S\left(h_{o}\left(t\right),r\right)q_{p}\left(t,r\right)\;dr+I_{s_{p}}\left(t\right)\int_{0}^{+\infty }\frac{\partial S}{\partial h}\left(I_{o}\left(t\right),r\right)q_{o}\left(t,r\right)\;dr, \end{array} \end{array}$$

we obtain

$$\begin{array}{c} \begin{array}{c} \int_{0}^{+\infty }(\frac{\partial }{\partial t}Z_{q}\left(t,r\right)+\frac{\partial }{\partial r}Z_{q}\left(t,r\right)-S\left(h_{o}\left(t\right),r\right)(Z_{q}\left(t,r\right)-Z_{q}\left(t,0\right)-\frac{J_{s}}{\tau_{s}}Z_{I_{s}}\left(t\right)))q_{p}\left(t,r\right)\;dr\\ +I_{p_{s}}\left(t\right)(\frac{d}{dt}Z_{I_{s}}\left(t\right)-\frac{1}{\tau_{s}}Z_{I_{s}}\left(t\right)-\int_{0}^{+\infty }(Z_{q}\left(t,r\right)-Z_{q}\left(t,0\right)-\frac{J_{s}}{\tau_{s}}Z_{I_{s}}\left(t\right))\frac{\partial S}{\partial h}\left(h_{o}\left(t\right),r\right)q_{o}\left(t,r\right)\;dr)=0. \end{array} \end{array}$$

Since this is true for every perturbation, the PRC must solve

$$\begin{array}{c} \begin{array}{c} -\frac{\partial }{\partial t}Z_{q}\left(t,r\right)-\frac{\partial }{\partial r}Z_{q}\left(t,r\right)=-S\left(h_{o}\left(t\right),r\right)[Z_{q}\left(t,r\right)-Z_{q}\left(t,0\right)-\frac{J_{s}}{\tau_{s}}Z_{I_{s}}\left(t\right)], \end{array} \end{array}$$ (10)

and

$$\begin{array}{c} \begin{array}{c} -\frac{d}{dt}Z_{I_{s}}\left(t\right)=-\frac{1}{\tau_{s}}Z_{I_{s}}\left(t\right)-\int_{0}^{+\infty }[Z_{q}\left(t,r\right)-Z_{q}\left(t,0\right)-\frac{J_{s}}{\tau_{s}}Z_{I_{s}}\left(t\right)]\frac{\partial S}{\partial h}\left(h_{o}\left(t\right),r\right)q_{o}\left(t,r\right)\;dr. \end{array} \end{array}$$ (11)

### Normalization condition

The adjoint equation being linear, its solution is unique under a normalization condition. In what follows we check that

$$\begin{array}{c} \begin{array}{c} \frac{d}{dt}\langle (\begin{array}{c} Z_{q}\\ Z_{I_{s}} \end{array}),(\begin{array}{c} \frac{\partial }{\partial t}q_{o}\\ \frac{d}{dt}I_{s_{o}} \end{array});t\rangle =0. \end{array} \end{array}$$

The computations that follow give rise to long mathematical expressions. We thus drop the function variables. After algebraic manipulations, we find that the above condition is equivalent to

$$\begin{array}{c} \begin{array}{c} \int_{0}^{+\infty }\frac{\partial }{\partial t}Z_{q}\frac{\partial }{\partial t}q_{o}\;dr+\frac{d}{dt}Z_{I_{s}}\frac{d}{dt}I_{s_{o}}+\int_{0}^{+\infty }\frac{\partial }{\partial t}Z_{q}\frac{\partial }{\partial t}(-\frac{\partial }{\partial r}q_{o}-S_{o}q_{o})\;dr\\ +\frac{d}{dt}Z_{I_{s}}\frac{\partial }{\partial t}(-\frac{1}{\tau_{s}}I_{s_{o}}+\frac{J_{s}}{\tau_{s}}A_{o})=0. \end{array} \end{array}$$

where we have introduced the new notations:

$$\begin{array}{c} A_{o}≔\int_{0}^{+\infty }S_{o}q_{o}\;dr\;S_{o}≔S\left(h_{o}\left(t\right),r\right). \end{array}$$

Now developing, we get

$$\begin{array}{c} \begin{array}{cc} & \int_{0}^{+\infty }\frac{\partial }{\partial t}Z_{q}\frac{\partial }{\partial t}(-\frac{\partial }{\partial r}q_{o}-S_{o}q_{o})\;dr\\ = & \int_{0}^{+\infty }\frac{\partial }{\partial t}Z_{q}(-\frac{\partial }{\partial r}\frac{\partial }{\partial t}q_{o}-S_{o}\frac{\partial }{\partial t}q_{o}-\frac{\partial S_{o}}{\partial h}q_{o}\frac{d}{dt}I_{s_{o}})\;dr\\ = & \int_{0}^{+\infty }\frac{\partial }{\partial t}\frac{\partial }{\partial r}Z_{q}\frac{\partial }{\partial t}q_{o}-Z_{q}S_{o}\frac{\partial }{\partial t}q_{o}-Z_{q}\frac{\partial S_{o}}{\partial h}q_{o}\frac{d}{dt}I_{s_{o}}\;dr-{\left[Z_{q}\frac{\partial }{\partial t}q_{o}\right]}_{0}^{+\infty }\\ = & \int_{0}^{+\infty }\frac{\partial }{\partial t}\frac{\partial }{\partial r}Z_{q}\frac{\partial }{\partial t}q_{o}-Z_{q}S_{o}\frac{\partial }{\partial t}q_{o}-Z_{q}\frac{\partial S_{o}}{\partial h}q_{o}\frac{d}{dt}I_{s_{o}}\;dr+Z_{q}\left(t,0\right)\frac{d}{dt}A_{o}. \end{array} \end{array}$$

We now use the fact that

$$\begin{array}{c} \begin{array}{c} \frac{d}{dt}A_{o}=\int_{0}^{+\infty }S_{o}\frac{\partial }{\partial t}q_{o}\;dr+\int_{0}^{+\infty }\frac{\partial S_{o}}{\partial h}q_{o}\frac{d}{dt}I_{s_{o}}\;dr. \end{array} \end{array}$$

Using this expression, we get that

$$\begin{array}{c} \begin{array}{cc} \int_{0}^{+\infty }\frac{\partial }{\partial t}Z_{q}\frac{\partial }{\partial t}(-\frac{\partial }{\partial r}q_{o}-S_{o}q_{o}) & \;dr+\frac{d}{dt}Z_{I_{s}}\frac{d}{dt}(-\frac{1}{\tau_{s}}I_{s_{o}}+\frac{J_{s}}{\tau_{s}}A_{o})\\ =\int_{0}^{+\infty }\frac{\partial }{\partial t} & q_{o}(\frac{\partial }{\partial r}Z_{q}-S_{o}Z_{q}+Z_{q}\left(t,0\right)S_{o}+\frac{J_{s}}{\tau_{s}}S_{o}Z_{I_{s}})\;dr\\ & +\frac{d}{dt}I_{o}(Z_{I_{s}}/\tau_{s}-\int_{0}^{+\infty }\frac{\partial S_{o}}{\partial h}q_{o}[Z_{q}-Z_{q}\left(t,0\right)-\frac{J_{s}}{\tau_{s}}Z_{I_{s}}]\;dr). \end{array} \end{array}$$

Putting everything together, we arrive to

$$\begin{array}{c} \begin{array}{cc} \frac{d}{dt}[\int_{0}^{+\infty }Z_{q}\frac{\partial }{\partial t}q_{o}\;dr & +Z_{I_{s}}\frac{d}{dt}I_{s_{o}}]\\ =\int_{0}^{+\infty }\frac{\partial }{\partial t} & q_{o}(\frac{\partial }{\partial t}Z_{q}+\frac{\partial }{\partial r}Z_{q}-S_{o}Z_{q}+Z_{q}\left(t,0\right)S_{o}+\frac{J_{s}}{\tau_{s}}S_{o}Z_{I_{s}})\;dr\\ & +\frac{d}{dt}I_{s_{o}}(\frac{d}{dt}Z_{I_{s}}-Z_{I_{s}}/\tau_{s}-\int_{0}^{+\infty }\frac{\partial S_{o}}{\partial h}q_{o}[Z_{q}-Z_{q}\left(t,0\right)-\frac{J_{s}}{\tau_{s}}Z_{I_{s}}]\;dr). \end{array} \end{array}$$

We now remind that the adjoint system is given by

$$\begin{array}{c} \begin{array}{c} -\frac{\partial }{\partial t}Z_{q}-\frac{\partial }{\partial r}Z_{q}=-S_{o}Z_{q}+Z_{q}\left(t,0\right)S_{o}+\frac{J_{s}}{\tau_{s}}S_{o}Z_{I_{s}}, \end{array} \end{array}$$

and

$$\begin{array}{c} \begin{array}{c} -\frac{d}{dt}Z_{I_{s}}-Z_{I_{s}}/\tau_{s}-\int_{0}^{+\infty }\frac{\partial S_{o}}{\partial h}q_{o}[Z_{q}-Z_{q}\left(t,0\right)-\frac{J_{s}}{\tau_{s}}Z_{I_{s}}]\;dr, \end{array} \end{array}$$

we therefore arrive to

$$\begin{array}{c} \frac{d}{dt}[\int_{0}^{+\infty }Z_{q}\frac{\partial }{\partial t}q_{o}\;dr+Z_{I_{s}}\frac{d}{dt}I_{s_{o}}]=0. \end{array}$$

The mPRC will be the unique solution satisfying the normalization condition:

$$\begin{array}{c} \int_{0}^{+\infty }Z_{q}\frac{\partial }{\partial t}q_{o}\;dr+Z_{I_{s}}\frac{d}{dt}I_{s_{o}}=\frac{2\pi }{T}, \end{array}$$

where *T* is nothing but the period of the oscillation.

### Numerical procedure

The mean-field Eq (8) can be readily integrated. We denote

$$\begin{array}{c} r_{j}=j\Delta t,\;\forall j>0,\;t_{n}=n\Delta t,\;\forall n>0, \end{array}$$

the discretization space/time variables, and

$$\begin{array}{c} q_{j}^{n}≔q(t^{n},r_{j}),\;S_{j}^{n}≔S(h^{n},r_{j}),\\ h^{n}≔h\left(t^{n}\right),\;I_{ext}^{n}≔I_{ext}\left(t^{n}\right)\;I_{s}^{n}≔I_{s}\left(t^{n}\right), \end{array}$$

the corresponding solution at the discretized points. Although theoretically *r* ∈ [0, ∞), for numerical purposes we need to truncate *r*. We have observed that the numerical methodology works as long as we truncate *r* at a value *r*_*max*_ large enough such that the whole population has produced a spike so *q*(*t*, *r*) → 0 for *r* > *r*_*max*_. We have observed *r*_*max*_ ≈ 1.25*T*_*ref*_ to be a good estimate.

Considering the initial state to be given, the mean-field Eq (8) can be numerically solved along the characteristic curves. On the characteristics, the dynamics reduce to a nonlinear differential equation that can be integrated with the following first order numerical scheme:

$$\begin{array}{c} \left\{ \begin{array}{cc} & q_{j+1}^{n+1}=q_{j}^{n}-\Delta tS_{j}^{n}q_{j}^{n}\\ & I_{s}^{n+1}=I_{s}^{n}+\Delta t(-I_{s}^{n}/\tau_{s}+J_{s}A^{n}/\tau_{s})\\ & q_{1}^{n+1}=A^{n+1}\\ & A^{n+1}=\Delta t\sum_{k\geq 1}S_{j}^{n+1}q_{j}^{n+1}\\ & h^{n+1}=I_{ext}^{n+1}+I_{s}^{n+1}. \end{array}\right. \end{array}$$ (12)

The proposed numerical scheme (12) is thus well defined and produces results in excellent agreement with simulations of the full network.

Using procedure (12) we find solutions of period *T* = *M*Δ*t* for the mean mean-field Eq (8) which we denote as $\bar{q}\left(t,r\right)$ and ${\bar{I}}_{s}\left(t,r\right)$. Next, we use the solutions $\bar{q}\left(t,r\right)$ and ${\bar{I}}_{s}\left(t,r\right)$ for solving the adjoint system (10) and (11).

Since the solution of the adjoint equation has an opposite stability with respect to the mean-field, we must integrate it backwards in time. We denote

$$\begin{array}{c} Z_{q_{j}}^{n}≔Z_{q}(t^{n},r_{j}),\;Z_{I_{s}}^{n}≔Z_{I_{s}}\left(t^{n}\right),\;{\bar{S}}_{j}^{n}≔S({\bar{h}}^{n},r_{j})\\ \;\partial {\bar{S}}_{j}^{n}≔\frac{\partial S}{\partial h}({\bar{h}}^{n},r_{j}),\;{\bar{h}}^{n}=I_{ext}^{n}+{\bar{I}}_{s}^{n}. \end{array}$$

Considering the end state to be given, the adjoint system (10) and (11) can be once again numerically solved along the characteristic curves. On the characteristics, the dynamics of the adjoint system (10) and (11) reduce to a linear differential equation that can be integrated with the following backward first order numerical scheme:

$$\begin{array}{c} \left\{ \begin{array}{cc} & Z_{q_{j-1}}^{n-1}=Z_{q_{j}}^{n}-\Delta t{\bar{S}}_{j}^{n}[Z_{q_{j}}^{n}-Z_{q_{1}}^{n}-J_{s}Z_{I_{s}}^{n}/\tau_{s}]\\ & Z_{q_{l}}^{n-1}=Z_{q_{l-1}}^{n-1}\;\;\;\;\;\;\;\;\;\;\;\;\text{for}\;l=\text{max}\left(j\right)\\ & Z_{I_{s}}^{n-1}=Z_{I_{s}}^{n}-\Delta t(Z_{I_{s}}^{n}/\tau_{s}+\sum_{k\geq 1}\partial {\bar{S}}_{k}^{n}[Z_{q_{k}}^{n}-Z_{q_{1}}^{n}-J_{s}Z_{I_{s}}^{n}/\tau_{s}]{\bar{q}}_{k}^{n}\Delta t). \end{array}\right. \end{array}$$ (13)

The proposed numerical scheme (13) is once again well defined and produces *T* periodic solutions ${\bar{Z}}_{q}\left(t,r\right)$ and ${\bar{Z}}_{I_{s}}\left(t\right)$ matching the PRC obtained by the direct perturbation method (see the main text). Next, we remark some numerical recipes which enhance the stability (and thus the convergence) of the procedure in (13). First, we iterate the scheme (13) over the periodic solutions $\bar{q}\left(t,r\right)$ and ${\bar{I}}_{s}\left(t,r\right)$ (recall ${\bar{q}}_{k}^{n+M}={\bar{q}}_{k}^{n}$). We also recommend computing the integral in (11) (that is, the sum for $Z_{I_{s}}^{n-1}$ in (13)) by using precise integration routines such as the trapezoidal rule or the Simpson’s method. Finally, since the procedure in (13) is based on backwards integration, it does not provide the value of *Z*_*q*_(*t*_*n*_, *r*_*j*_) at *r* = max(*r*_*j*_). This value can be obtained by simple extrapolation (as we propose in (13)) or by using accurate extrapolation routines taking into account a larger set of values of *Z*_*q*_(*t*_*n*_, *r*_*j*_). We remark that, although the smaller the Δ*t* value the higher the accuracy of solutions, the usage of the above mentioned recipes generates very precise results for time steps Δ*t* ≈ 0.005. We also remark that the procedure on (13) relies on the periodic solutions $\gamma \left(t,r\right)=\{\bar{q}\left(t,r\right),{\bar{I}}_{s}\left(t\right)\}$ obtained from (12). To ensure stability of (13), it is necessary to consider integration times large enough so the periodic solutions *γ*(*t*, *r*) are accurate enough (that is, ‖*γ*(*t*, *r*) − *γ*(*t* + *T*, *r*)‖ ≈ 0, with ‖⋅‖ the Euclidian norm).

### Coupled networks and the phase equation

Considering two bidirectionally delayed coupled networks where the coupling is made via long projections from one network to another, the whole system reduces to a set of coupled partial differential equation. For the first network, we have

$$\begin{array}{c} \begin{array}{c} \frac{\partial }{\partial t}q_{1}+\frac{\partial }{\partial r}q_{1}=-S\left(h_{1}\left(t\right),r\right)q_{1}, \end{array} \end{array}$$

and

$$\begin{array}{c} \begin{array}{c} \frac{\partial }{\partial t}q_{2}+\frac{\partial }{\partial r}q_{2}=-S\left(h_{2}\left(t\right),r\right)q_{2}. \end{array} \end{array}$$

The boundary conditions are given by

$$\begin{array}{c} q_{1}\left(t,0\right)=A_{1}\left(t\right)=\int_{0}^{+\infty }S\left(h_{1}\left(t\right),r\right)q_{1}\left(t,r\right)\;dr, \end{array}$$

and

$$\begin{array}{c} q_{2}\left(t,0\right)=A_{2}\left(t\right)=\int_{0}^{+\infty }S\left(h_{2}\left(t\right),r\right)q_{2}\left(t,r\right)\;dr. \end{array}$$

The total input current is still given by

$$\begin{array}{c} h_{1}=I_{ext}+I_{s_{1}},\;h_{2}=I_{ext}+I_{s_{2}}, \end{array}$$

the synaptic current *I*_*s*_(*t*) is computed as

$$\begin{array}{c} \begin{array}{c} \tau_{s}\frac{d}{dt}I_{s_{1}}\left(t\right)=-I_{s_{1}}\left(t\right)+J_{s}A_{1}\left(t\right)+\varepsilon G_{s}A_{2}\left(t-d\right), \end{array} \end{array}$$

and

$$\begin{array}{c} \begin{array}{c} \tau_{s}\frac{d}{dt}I_{s_{2}}\left(t\right)=-I_{s_{2}}\left(t\right)+J_{s}A_{2}\left(t\right)+\varepsilon G_{s}A_{1}\left(t-d\right). \end{array} \end{array}$$

Here *G*_*s*_ denotes the connectivity strength across circuits, the parameter *ε* emphasises the weak coupling assumption, and the parameter *d* is the conduction delay between the two networks. Assuming that the two networks are oscillating and placing our study within the framework of weakly coupled oscillators, that is, if we assume that

ε<<1,

we can reduce the bidirectionally delayed-coupled neural circuits description to a single phase equation:

$$\begin{array}{c} \frac{d}{dt}\theta \left(t\right)=G\left(\theta \left(t\right)\right). \end{array}$$

Here *θ*(*t*) is the phase difference (or phase lag) between the circuits and the *G*-function is the odd part of the shifted interaction function (the *H*-function), see [56] for instance:

$$\begin{array}{c} G(\theta )=H(\theta -d)-H(-\theta -d), \end{array}$$

with *d*, the time delay between the two circuits. In our case, the interaction function is mathematically described as

$$\begin{array}{c} \begin{array}{cc} H(\theta )= & \frac{\varepsilon G_{s}}{T}\int_{0}^{T}Z_{I}\left(s\right)A\left(s-\theta \right)\;ds \end{array} \end{array}$$

where *T* is the oscillation period.

### Excitatory-inhibitory network

In the thermodynamic limit the network description of a pair of excitatory-inhibitory populations reduces to a set of coupled partial differential equations. Denoting *q*_*e*_(*t*, *r*) the probability density for a excitatory neuron to have at time *t* an age *r*, and *q*_*i*_(*t*, *r*) for the inhibitory population, the evolution of the density profiles evolve according to the continuity equations:

$$\begin{array}{c} \begin{array}{c} \frac{\partial }{\partial t}q_{e}+\frac{\partial }{\partial r}q_{e}=-S_{e}\left(h_{e}\left(t\right),r\right)q_{e}, \end{array} \end{array}$$

and

$$\begin{array}{c} \begin{array}{c} \frac{\partial }{\partial t}q_{i}+\frac{\partial }{\partial r}q_{i}=-S_{i}\left(h_{i}\left(t\right),r\right)q_{i}. \end{array} \end{array}$$

The boundary conditions are given by

$$\begin{array}{c} q_{e}\left(t,0\right)=A_{e}\left(t\right)=\int_{0}^{+\infty }S_{e}\left(h_{e}\left(t\right),r\right)q_{e}\left(t,r\right)\;dr, \end{array}$$

and

$$\begin{array}{c} q_{i}\left(t,0\right)=A_{i}\left(t\right)=\int_{0}^{+\infty }S_{i}\left(h_{i}\left(t\right),r\right)q_{i}\left(t,r\right)\;dr. \end{array}$$

The total input current is still given by

$$\begin{array}{c} h_{e}=I_{ext}^{e}+I_{s_{e}},\;h_{i}=I_{ext}^{i}+I_{s_{i}}, \end{array}$$

the synaptic current *I*_*s*_(*t*) is computed as

$$\begin{array}{c} \begin{array}{c} I_{s_{e}}=J_{ee}\kappa *A_{e}-J_{ei}\kappa *A_{i},\;I_{s_{i}}=J_{ie}\kappa *A_{e}-J_{ii}\kappa *A_{i}. \end{array} \end{array}$$

We can now define the corresponding bi-linear form:

$$\begin{array}{c} \langle (\begin{array}{c} q_{e_{1}}\\ I_{e_{1}}\\ q_{i_{1}}\\ I_{i_{1}} \end{array}),(\begin{array}{c} q_{e_{2}}\\ I_{e_{2}}\\ q_{i_{2}}\\ I_{i_{2}} \end{array});t\rangle =\int_{0}^{+\infty }q_{e_{1}}q_{e_{2}}\;dr+I_{e_{1}}I_{e_{2}}+\int_{0}^{+\infty }q_{i_{1}}q_{i_{2}}\;dr+I_{i_{1}}I_{i_{2}}. \end{array}$$

Assuming to be known the periodic solution, $\left(q_{e_{o}},I_{s_{e_{o}}}\right)$ and $\left(q_{i_{o}},I_{s_{i_{o}}}\right)$, computations similar to what is presented within the adjoint section, we find that the PRC must solves:

$$\begin{array}{c} \begin{array}{c} -\frac{\partial }{\partial t}Z_{q_{e}}-\frac{\partial }{\partial r}Z_{q_{e}}=-S_{e}\left(h_{e_{o}}\left(t\right),r\right)[Z_{q_{e}}-Z_{q_{e}}\left(t,0\right)-\frac{J_{ee}}{\tau_{s}}Z_{I_{s_{e}}}+\frac{J_{ei}}{\tau_{s}}Z_{I_{s_{i}}}], \end{array} \end{array}$$

and

$$\begin{array}{c} \begin{array}{cc} -\frac{d}{dt}Z_{I_{s_{e}}}=-\frac{1}{\tau_{s}}Z_{I_{s_{e}}} & -\int_{0}^{+\infty }[Z_{q_{e}}-Z_{q_{e}}\left(t,0\right)-\frac{J_{ee}}{\tau_{s}}Z_{I_{s_{e}}}]\frac{\partial S_{e}}{\partial h_{e}}\left(h_{e_{o}}\left(t\right),r\right)q_{e_{o}}\;dr\\ & -\int_{0}^{+\infty }[Z_{q_{i}}-Z_{q_{i}}\left(t,0\right)+\frac{J_{ei}}{\tau_{s}}Z_{I_{s_{i}}}]\frac{\partial S_{i}}{\partial h_{i}}\left(h_{i_{o}}\left(t\right),r\right)q_{i_{o}}\;dr. \end{array} \end{array}$$

similarly

$$\begin{array}{c} \begin{array}{c} -\frac{\partial }{\partial t}Z_{q_{i}}-\frac{\partial }{\partial r}Z_{q_{i}}=-S_{i}\left(h_{i_{o}}\left(t\right),r\right)[Z_{q_{i}}-Z_{q_{i}}\left(t,0\right)-\frac{J_{ie}}{\tau_{s}}Z_{I_{s_{e}}}+\frac{J_{ii}}{\tau_{s}}Z_{I_{s_{i}}}], \end{array} \end{array}$$

and

$$\begin{array}{c} \begin{array}{cc} -\frac{d}{dt}Z_{I_{s_{i}}}=-\frac{1}{\tau_{s}}Z_{I_{s_{i}}} & -\int_{0}^{+\infty }[Z_{q_{e}}-Z_{q_{e}}\left(t,0\right)-\frac{J_{ie}}{\tau_{s}}Z_{I_{s_{e}}}]\frac{\partial S_{e}}{\partial h_{e}}\left(h_{e_{o}}\left(t\right),r\right)q_{e_{o}}\;dr\\ & -\int_{0}^{+\infty }[Z_{q_{i}}-Z_{q_{i}}\left(t,0\right)+\frac{J_{ii}}{\tau_{s}}Z_{I_{s_{i}}}]\frac{\partial S_{i}}{\partial h_{i}}\left(h_{i_{o}}\left(t\right),r\right)q_{i_{o}}\;dr. \end{array} \end{array}$$

Incoming perturbation should get through the synapse, $Z_{I_{s}}$ should be interpreted as the mPRC of the macroscopic oscillation. Two PRCs can therefore be defined $Z_{I_{s_{e}}}$ and $Z_{I_{s_{i}}}$ at the same time. The PRC defined by $Z_{I_{s_{e}}}$ corresponds to excitatory input arriving upon the E-cells, while $Z_{I_{s_{i}}}$ corresponds to excitatory input arriving upon the I-cells.

The normalisation condition is now given by:

$$\begin{array}{c} \int_{0}^{+\infty }Z_{q_{e}}\frac{\partial }{\partial t}q_{o_{e}}\;dr+Z_{I_{s_{e}}}\frac{d}{dt}I_{s_{o_{e}}}+\int_{0}^{+\infty }Z_{q_{i}}\frac{\partial }{\partial t}q_{o_{i}}\;dr+Z_{I_{s_{i}}}\frac{d}{dt}I_{s_{o_{i}}}=\frac{2\pi }{T}, \end{array}$$

with again *T* the oscillation period.

### Complementary approach for conductance-based models

In this section, we recall another framework in use in Computational Neuroscience. In [44], the authors have constructed a mapping between voltage-based models and the von Foerster equation. For instance, starting withe the integrate-and-fire model, see [52]:

$$\begin{array}{c} C\frac{dv}{dt}=-G\left(v-V_{L}\right)+h\left(t\right)+\sigma \eta \left(t\right), \end{array}$$

together with a threshold *V*_*T*_ and a reset *V*_*r*_ to account for an action potential. Here *h*(*t*) is the stimulus, *C*, the capacitance, *G*, the conductance, *V*_*L*_, the reversal potential, and *σ*, the scaling of the white noise *η*. It has been shown that this is equivalent to the following equations [52]:

$$\begin{array}{c} \begin{array}{c} \frac{\partial }{\partial t}q\left(t,r\right)+\frac{\partial }{\partial r}q\left(t,r\right)=-S\left(u\left(t,r\right),\dot{u}\left(t,r\right)\right)q\left(t,r\right), \end{array} \end{array}$$

where *u*(*t*, *r*) is given by

$$\begin{array}{c} \begin{array}{c} C(\frac{\partial }{\partial t}u\left(t,r\right)+\frac{\partial }{\partial r}u\left(t,r\right))=-G\left(u\left(t,r\right)-V_{L}\right)+h\left(t\right). \end{array} \end{array}$$

The boundary conditions of the two partial differential equations are given by

$$\begin{array}{c} q\left(t,0\right)=\int_{0}^{+\infty }S\left(u\left(t,r\right),\dot{u}\left(t,r\right)\right)q\left(t,r\right)\;dr, \end{array}$$

and for *u*, it is given by

$$\begin{array}{c} u\left(t,0\right)=V_{r}. \end{array}$$

Defining the corresponding bi-linear form:

$$\begin{array}{c} \langle (\begin{array}{c} q\\ I\\ u \end{array}),(\begin{array}{c} Z_{q}\\ Z_{I}\\ Z_{u} \end{array});t\rangle =\int_{0}^{+\infty }qZ_{q}\;dr+IZ_{I}+\int_{0}^{+\infty }uZ_{u}\;dr, \end{array}$$

and assuming to be known the periodic solution, $\left(q_{o},I_{s_{o}},u_{o}\right)$, computations similar to what is presented within the adjoint section, we find that the mPRC must solves:

$$\begin{array}{c} \begin{array}{c} -\frac{\partial }{\partial t}Z_{q}-\frac{\partial }{\partial r}Z_{q}=-S_{o}[Z_{q}-Z_{q}\left(t,0\right)-\frac{J}{\tau_{s}}Z_{I_{s}}], \end{array} \end{array}$$

and

$$\begin{array}{c} \begin{array}{c} -\frac{\partial }{\partial t}Z_{u}-\frac{\partial }{\partial r}Z_{u}=-\frac{G}{C}Z_{u}+[\frac{G}{C}\frac{\partial S_{o}}{\partial u}q_{o}-\frac{\partial S_{o}}{\partial \dot{u}}q_{o}][Z_{q}-Z_{q}\left(t,0\right)-\frac{J}{\tau_{s}}Z_{I_{s}}], \end{array} \end{array}$$

and

$$\begin{array}{c} \begin{array}{cc} -\frac{d}{dt}Z_{I_{s}}=-\frac{1}{\tau_{s}}Z_{I_{s}} & -\int_{0}^{+\infty }[\frac{1}{C}Z_{q}-\frac{1}{C}Z_{q}\left(t,0\right)-\frac{J}{\tau_{s}}Z_{I_{s}}]\frac{\partial S_{o}}{\partial \dot{u}}q_{o}-\frac{1}{C}Z_{u}\;dr,\; \end{array} \end{array}$$

where we have used the notation:

$$\begin{array}{c} \begin{array}{c} S_{o}≔S\left(u_{o}\left(t,r\right),{\dot{u}}_{o}\left(t,r\right)\right). \end{array} \end{array}$$

Incoming perturbation should get through the synapse, $Z_{I_{s}}$ should be interpreted as the mPRC of the macroscopic oscillation. The normalisation condition is now given by:

$$\begin{array}{c} \int_{0}^{+\infty }Z_{q}\frac{\partial }{\partial t}q_{o}\;dr+Z_{I_{s}}\frac{d}{dt}I_{s_{o}}+\int_{0}^{+\infty }Z_{u}\frac{\partial }{\partial t}u_{o}\;dr=\frac{2\pi }{T}, \end{array}$$

with again *T* the oscillation period.

## Supporting information

S1 Python script Python script to compute the solution of the mean-field equation and its associated adjoint. (ZIP)
