# Supplementary figures and images for: A framework for macroscopic phase-resetting curves for generalised spiking neural networks
Source: PLoS Comput Biol. 2022 Aug 1;18(8):e1010363. doi: 10.1371/journal.pcbi.1010363 (PMC9371324; doi:10.1371/journal.pcbi.1010363)

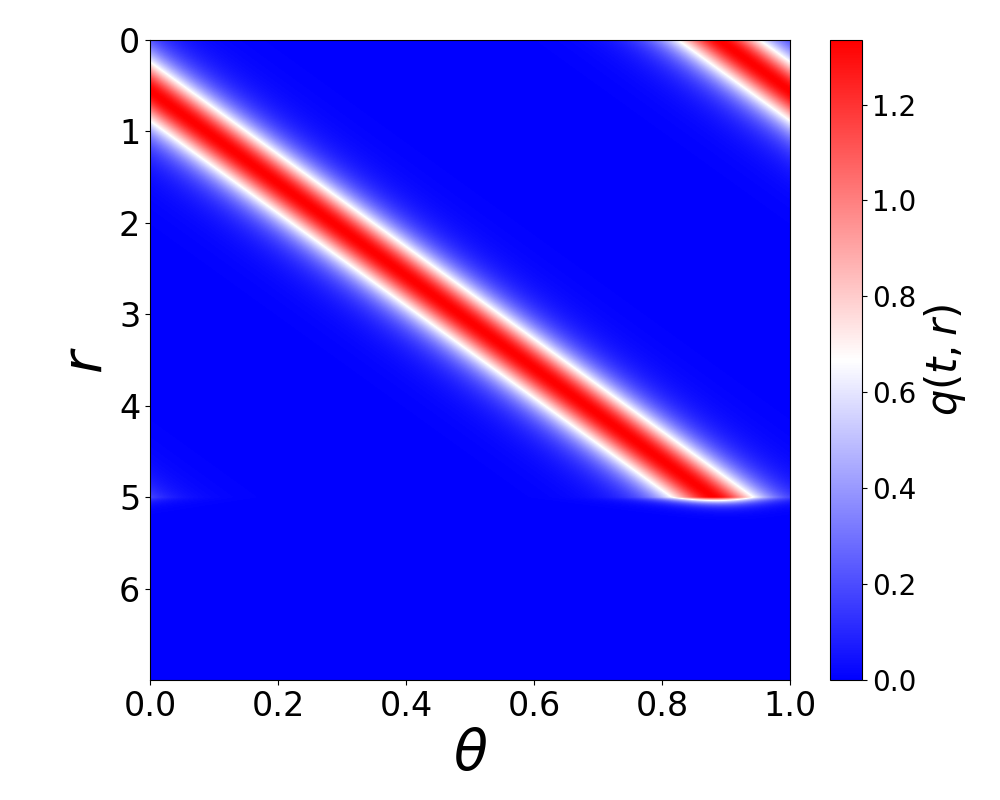

Supplement: S1 Python script — (ZIP) [file pcbi.1010363.s001.zip › gitHub/qCasA.png]

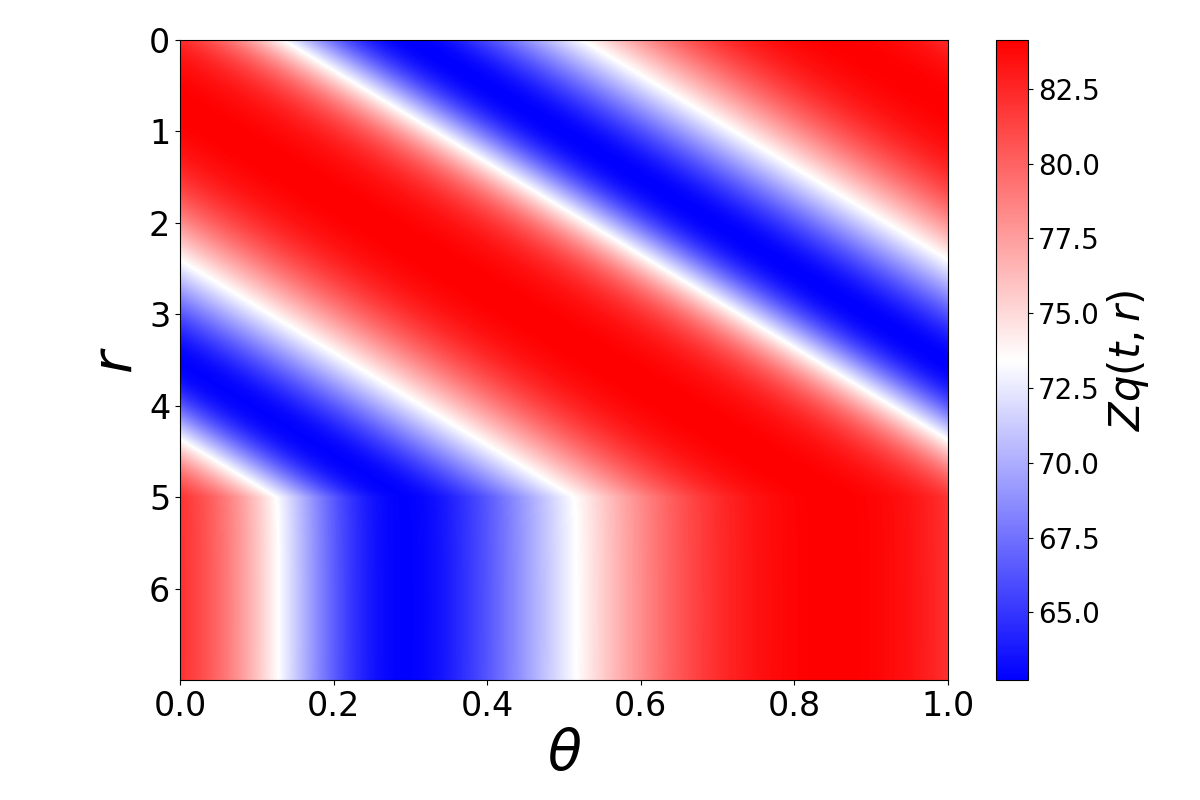

Supplement: S1 Python script — (ZIP) [file pcbi.1010363.s001.zip › gitHub/zqCasA.png]

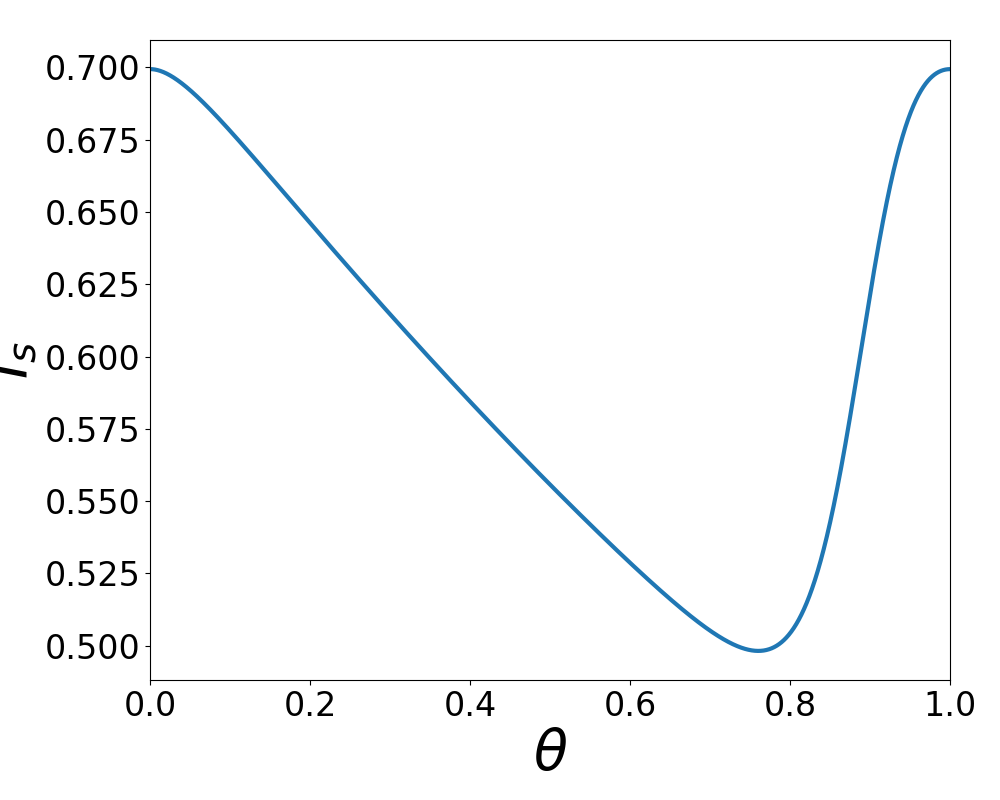

Supplement: S1 Python script — (ZIP) [file pcbi.1010363.s001.zip › gitHub/I_sCasA.png]

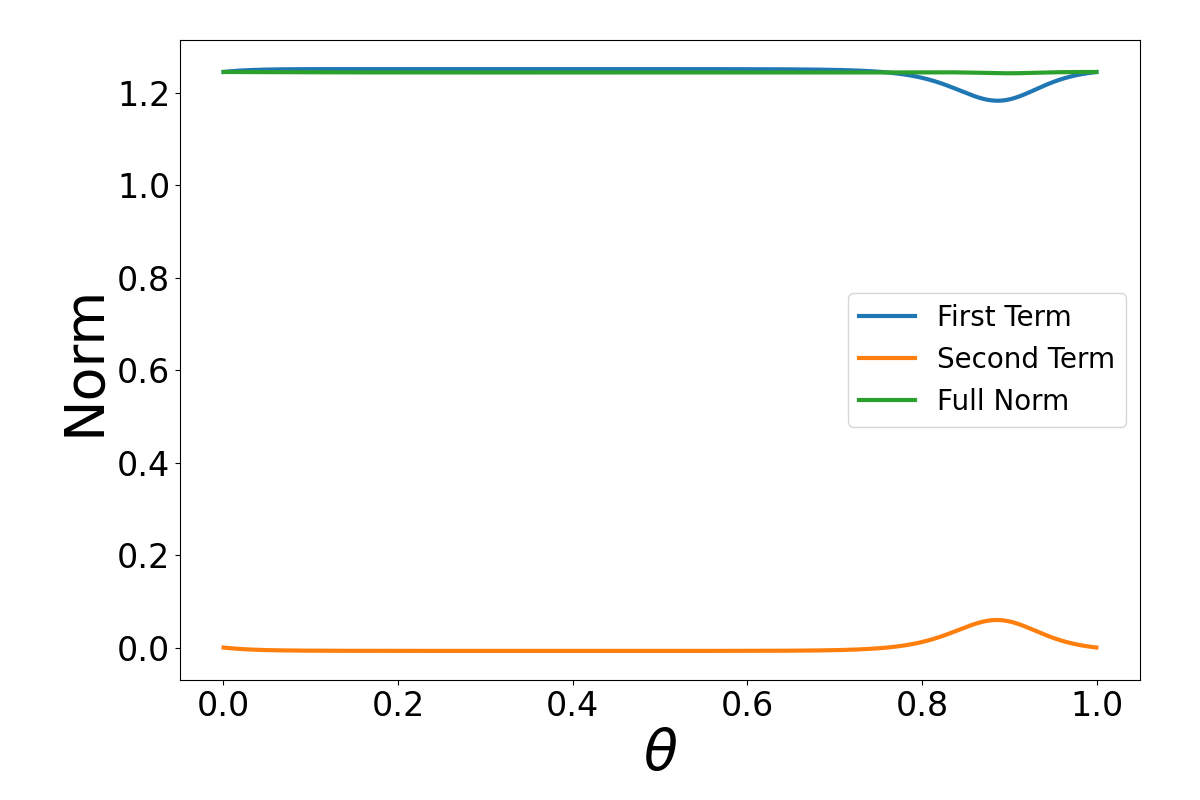

Supplement: S1 Python script — (ZIP) [file pcbi.1010363.s001.zip › gitHub/norm_sCasA.png]

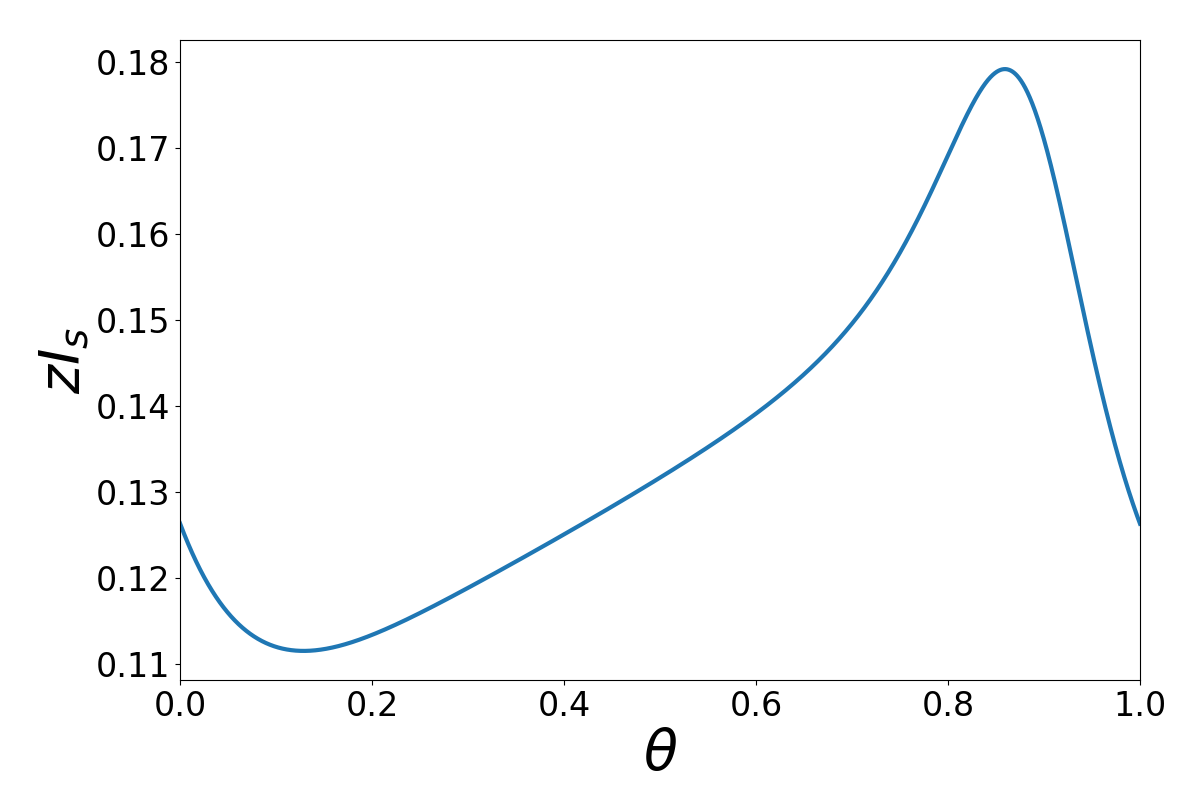

Supplement: S1 Python script — (ZIP) [file pcbi.1010363.s001.zip › gitHub/zI_sCasA.png]

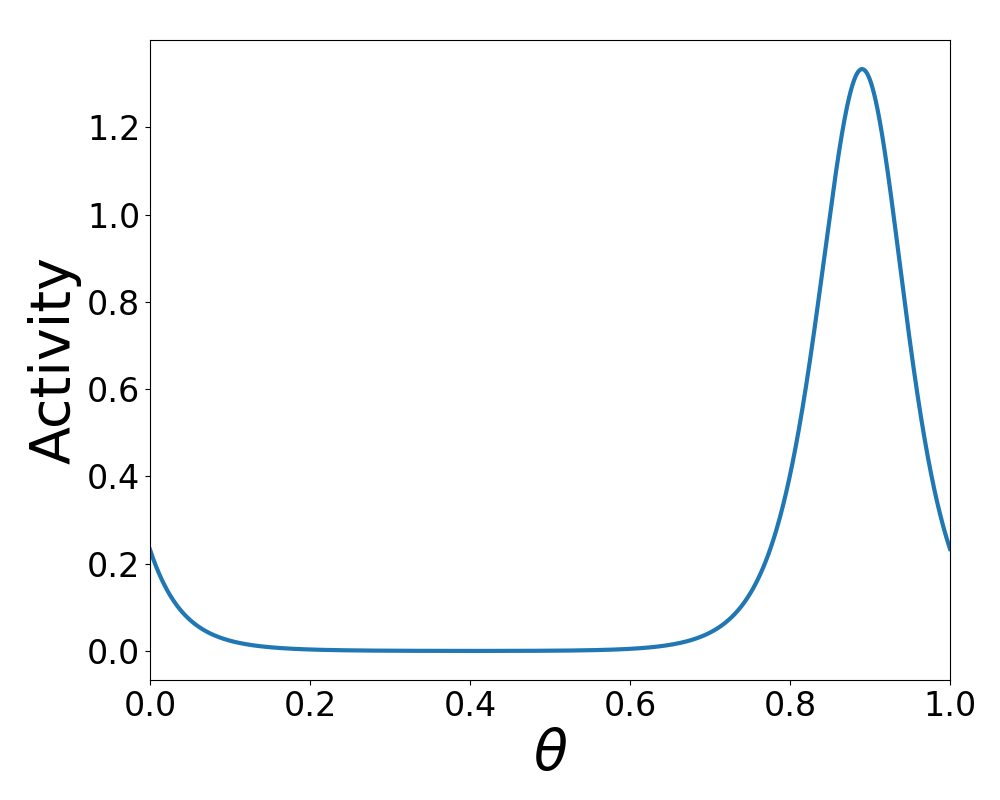

Supplement: S1 Python script — (ZIP) [file pcbi.1010363.s001.zip › gitHub/aCasA.png]
